# Effectiveness of a Mobile App Intervention for Preparing Preschool Children and Parents for Day Surgery: Randomized Controlled Trial

**DOI:** 10.2196/46989

**Published:** 2023-09-29

**Authors:** Heli Kerimaa, Mervi Hakala, Marianne Haapea, Hannu Vähänikkilä, Willy Serlo, Hong-Gu He, Tarja Pölkki

**Affiliations:** 1 Medical Research Center Oulu Oulu University Hospital and University of Oulu Oulu Finland; 2 Research Unit of Health Sciences and Technology University of Oulu Oulu Finland; 3 Oulu University Hospital Oulu Finland; 4 Research Service Unit Oulu University Hospital Oulu Finland; 5 Northern Finland Birth Cohorts Arctic Biobank, Infrastructure for Population Studies Faculty of Medicine, University of Oulu Oulu Finland; 6 Division of Pediatric Surgery, Oulu University Hospital, Oulu, Finland Oulu Finland; 7 Oulu University Unit of Clinical Medicine, University of Oulu Oulu Finland; 8 Alice Lee Centre for Nursing Studies, Yong Loo Lin School of Medicine National University of Singapore Singapore Singapore; 9 National University Health System Singapore Singapore

**Keywords:** anxiety, day surgery, fear, mobile app, pain, parents, preparation, preschool child, randomized controlled trial, stress, mobile phone

## Abstract

**Background:**

Day surgery allows families to return home quickly. Only a few approaches to preparing for day surgery have demonstrated how digital solutions can support families and children.

**Objective:**

This study aims to evaluate the effectiveness of a mobile app intervention on preschool children’s fear and pain and parents’ anxiety and stress in preparing children for day surgery.

**Methods:**

This study was conducted at the Pediatric Day Surgical Department of a university hospital in Finland between 2018 and 2020. Parents of children (aged 2-6 y) who were in a queue for elective day surgery were randomized into the intervention group (IG; n=36) and control group (CG; n=34). The CG received routine preparations, whereas the IG was prepared using a mobile app. Parents’ and children’s outcomes were measured using validated scales at 4 different points: at home (T1 and T4) and at the hospital (T2 and T3) before and after surgery. Group differences were analyzed using statistical methods suitable for the material.

**Results:**

Before surgery, parents in both groups experienced mild anxiety, which decreased after surgery. Parental anxiety did not differ between groups preoperatively (*P*=.78) or postoperatively (*P*=.63). Both groups had less anxiety at home after surgery compared with before. The IG showed a significant decrease (*P*=.003); the CG also improved (*P*=.002). Preoperatively at home, most parents in both groups experienced no stress or mild stress (*P*=.61). Preoperatively at the hospital, parents in both groups experienced mild stress; however, parents in the IG experienced more stress during this phase (*P*=.02). Parents in the IG experienced significantly less stress postoperatively than those in the CG (*P*=.05). Both groups showed decreased stress levels from before to after surgery (IG: *P*=.003; CG: *P*=.004) within each group. There were no significant differences in children’s pain levels between the groups and measurement points. This was observed before surgery at home (*P*=.25), before surgery at the hospital (*P*=.98), and after surgery at the hospital (*P*=.72). Children’s fear decreased more in the IG (*P*=.006) than in the CG (*P*=.44) comparing the phases before and after surgery at home. Fear did not differ between the IG and CG preoperatively at home (*P*=.20) or at the hospital (*P*=.59) or postoperatively at the hospital (*P*=.62) or at home (*P*=.81).

**Conclusions:**

The mobile app intervention did not reduce anxiety or pain. However, it was observed that parents in the IG experienced substantially heightened stress levels before surgery at the hospital, which decreased significantly after surgery at home. In addition, fear levels in children in the IG decreased over time, whereas no significant change was observed in the CG. These results are important for developing health care service chains and providing families with innovative and customer-oriented preparation methods.

**Trial Registration:**

ClinicalTrials.gov NCT03774303; https://classic.clinicaltrials.gov/ct2/show/NCT03774303

## Introduction

### Background

Day surgery affords families the possibility of a quick return home and to everyday life, usually within 1 day [1]. Worldwide, the number of day surgery procedures in children has increased significantly. In the United States, >5 million pediatric patients are estimated to participate in planned day surgeries or diagnostic procedures every year [2,3]. Other countries have also witnessed a growing number of pediatric patients in recent years [4]. As pediatric day surgery rates increase, parents need to be educated about their role in day surgery and what happens at different stages of the surgical process [4]. Their situation is rather complicated as parents play several roles in preparing their children for day surgery: they bear responsibility, make important decisions, and support the child through the process [5,6]. Therefore, it is understandable that parents often experience anxiety and stress [3,7,8], which are natural responses to a new, challenging situation [9]. Preschool children (aged 2-6 y) also find day surgery scary [10,11]. A child’s fear is often related to the unknown [11] and the worry about being separated from their parents [12,13]. The rapid development of information and communications technologies and the increased prevalence of smartphones have created new opportunities for using web-based or digital programs to prepare patients for day surgery [14,15]. The effectiveness of mobile app interventions for preschool children and parents has already been studied in children with chronic illnesses [16], adults [17,18], and health monitoring [19]. In the older age group (9-17 y), studies have shown that playing computer games before surgery can be effective in reducing children’s separation and presurgery anxiety levels [20].

Preparation for pediatric day surgery sets out to improve parents’ understanding of the surgery to be performed and improve cooperation between the child and health care personnel. According to earlier studies, approximately 50% to 70% of parents experience anxiety [21] and stress [22] about their child’s day surgery [23,24]. This anxiety may be transferred to the child, amplifying their feelings of pain and fear [3,7,8]. Parental anxiety and stress are caused by uncertainty, lack of control in a new situation [9], and various fears about pediatric surgery [5,6]. Parents’ lack of knowledge can lead them to experience guilt, ignorance, and separation anxiety and feel out of control [9]. Research has also shown that 65% to 80% of children experience anxiety or fear [25] before surgery [10,26,27]. Up to 30% of children also experience moderate or significant pain [28] as a result of day surgery [10,27,29].

This is a significant challenge for parents, who are responsible for preparing their children for day surgery [30] and addressing their concerns. Parents need to make important decisions and support their children [5,6], who may well be experiencing fear and anxiety [31], which can negatively affect both the surgery and the recovery from it [32]. The situation can be made even more challenging by a child’s active imagination, which is stronger at a younger age. A child cannot use abstract logical thinking [33], and the concept of day surgery may be difficult for them to understand [34]. Children who experience preoperative fear also typically experience more pain after surgery, need more medication, and recover more slowly [31,32,35]. Furthermore, a child’s age is inversely related to their parents’ experience of their day surgery, meaning that parents of younger children tend to experience more anxiety than parents of school-age children (7-16 y) [36].

### Prior Work

Previous research has found that insufficient preparation time is linked to higher levels of parental fear, anxiety, and stress [15]. The preparation of preschool children and their parents for day surgery should be well planned [37], sufficiently diverse [38], and timely [39], covering all the phases of perioperative care through to recovery at home [37,38]. There is an adequate existing knowledge base regarding the preparation of parents for day surgery [5,7,34], but little is known about the use and efficacy of mobile apps in doing this [40]. Liu et al [40] have suggested that the mobile app WeChat could be used as an efficient preparation method for parents whose children are undergoing surgery for a hernia. They found that using WeChat increased parental knowledge and reduced surgery cancellations [40]. To develop customer-oriented services for day surgery, it is important to study the usability of any app developed for different age groups.

Smartphones and their associated apps have become an integral part of everyday life. The World Health Organization [41] has stated that digitalization can help support human health and access to high-quality health services and improve the efficiency and sustainability of health systems. However, it is essential to understand which elements lead to the best improvements for both parents and children [42] if the content and cost-effectiveness of these solutions is to be optimized. To the best of our knowledge, the effectiveness of mobile app interventions in preparing preschool children and their families for day surgery has not been studied in any depth. Most previous studies have focused on older age groups and have generally described apps that target young people with long-term illnesses [16]. In addition, research into relevant mobile apps has usually focused on children’s pain and primarily concerned children aged >8 years [43]. According to Rantala et al [44], there is a research gap concerning how digital solutions can support families and children.

### Goal of This Study

The aim of this study was to evaluate the effectiveness of a mobile app intervention for preparing preschool children and their parents for pediatric day surgery. In this study, children’s pain and fear and parents’ anxiety and stress were measured at home and at the hospital before and after day surgery. The first hypothesis was that parents in the intervention group (IG) would show lower levels of anxiety and stress at home and at the hospital before and after the day surgery than parents in the control group (CG). The second hypothesis was that preschool children in the IG would show lower levels of pain and fear at home and at the hospital before and after the day surgery than preschool children in the CG.

## Methods

### Study Design

This study used a 2-armed randomized controlled trial design.

### Setting and Sample

#### Overview

This study was conducted between January 2018 and May 2020 at the Pediatric Day Surgical Department of a university hospital in Finland. Participants included the parents of preschool children (aged 2-6 y) who were due to undergo elective day surgery under general anesthesia. The other inclusion criteria are listed in Textbox 1. The mobile app was available for use for 3 to 4 weeks before the operation so that parents had sufficient time to use it to prepare for their child’s surgery.

**Study inclusion criteria.**

- Child: child eligible for day surgery
- Child age: children aged between 2 and 6 y
- Child surgery: testicular repairs (undescended testicle), repairs of foreskin stenosis, hernias, surgeries related to skin and subcutaneous tissues (excluding laser surgery), and orthopedics (eg, ganglia but excluding the removal of fixation material)
- Surgery type: elective surgery
- Risk classification: American Society of Anesthesiologists 1-2
- Food intake: >6 h of fasting
- Parents: parents present
- Back to home: discharge on the same day
- Anesthesia: general anesthesia
- Operating technique: not laparoscopy
- Pain management: premedicated analgesic as usual; local anesthesia at the end of the surgery to the operational area as usual
- Premedication: if needed
- Technology: parents with access to an Android or iOS phone, iPad, or an internet browser
- Other research-related delimitation: Finnish-speaking families only as the app was in Finnish; the time window was 3-4 wk before the operation so that the family had adequate time to prepare for the surgery

#### Sample Size Calculation

According to a study by Kain et al [35], 46% of all parents experience anxiety before surgery as measured by the State-Trait Anxiety Inventory (STAI). To determine our sample size, we used the findings from the study by Kain et al [35], which had an IG with a mean anxiety of 39.7 (SD 11.5) and a CG with a mean anxiety of 48.6 (SD 13.1). Our study focused on the primary outcome of parental anxiety as measured using the STAI scale. An independent-sample 1-tailed *t* test with α value of .05 and 80% power estimated that 50 participants would be required for the study, with 25 participants in each group (intervention and control). We adjusted the sample size to account for a potential 30% dropout rate. As a result, the final sample comprised 70 families with children, with 36 (51%) in the IG and 34 (49%) in the CG. A total of 70 parents were recruited and randomized [45].

#### Randomization, Allocation Concealment, and Blinding

**Figure 1:**
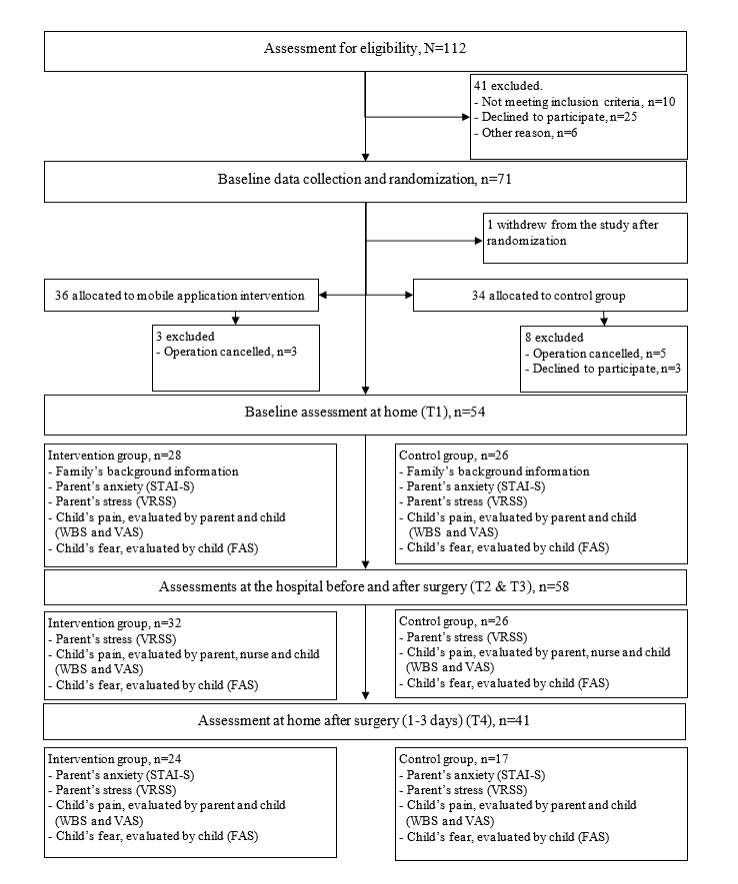
The CONSORT (Consolidated Standards of Reporting Trials) diagram for this study. FAS: Facial Affective Scale; PPPM: Parents’ Postoperative Pain Measure; STAI-S: State-Trait Anxiety Inventory; VAS; visual analogue scale; VRSS: Verbal Rating Scale for Stress; WBS: Wong-Baker Faces Pain Rating Scale.

The eligible participants were categorized into 5 strata according to the age of the child undergoing surgery (2, 3, 4, 5, and 6 y) and then randomized into each group at a 1:1 ratio. We used stratified simple randomization to keep the groups as similar as possible [46]. The researcher prepared 2 envelopes in advance, with one for children aged 2 years and the other for children up to the age of 6 years. A total of 10 notes were placed into each age group envelope, 5 of which were allocated to the IG, and the remaining 5 were allocated to the CG. Following ethical guidelines, after a telephone conversation with each set of parents, the researcher took one note from the envelope corresponding to the child’s age group to determine whether the family was allocated to the IG or the CG. Both the researcher and participants were unaware of which group they were allocated to before the study. A flowchart of the study is presented in Figure 1.

### Intervention

#### Developing and Piloting the Mobile App

**Figure 2:**
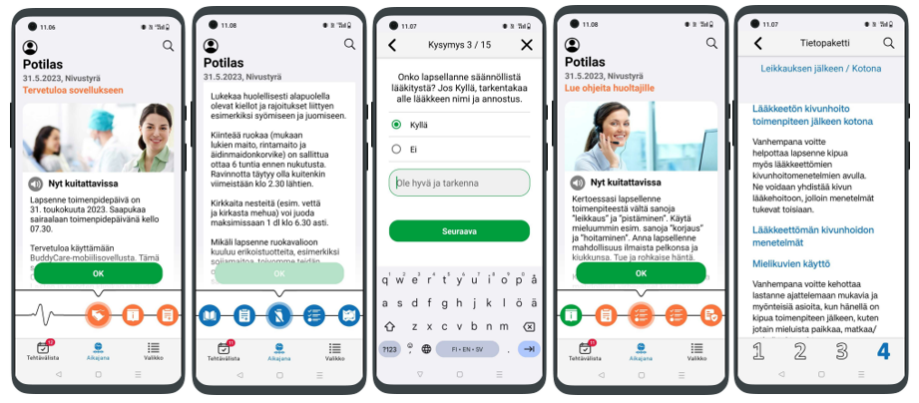
BuddyCare app screenshots.

A mobile app intervention that supports families and children before and after surgery was developed in collaboration with the children’s and adolescents’ hospital unit and a commercial company (Buddy Healthcare Ltd). This intervention was designed to relieve both children’s and their parents’ stress, anxiety, pain, and fear related to day surgery. The intervention was developed through multiprofessional collaboration considering expert input from nurses and physicians involved in pediatric day surgery. The development process also considered that each hospital, family, and child is unique. The information provided in the app was easy to understand using a simple timeline and incorporating the routine preparation material received by the CG. The purpose of the timeline was to inform users about the phases of the surgery and provide information to families at the relevant time (Figure 2). The app contained information for both parents and children presented in various formats, including video, photo, and written instructions. Contents included images of the day surgery unit, instructions for the surgery and pain care, information on how to get to the hospital and the ward, notifications, and the necessary forms. After registering on the app, users’ information was passed on to the hospital, allowing health care professionals (HCPs) to keep track of a family’s preparation for the surgery. The app also included a video describing the progression through surgery at the hospital (from arrival to discharge, along with information about some nonpharmacological pain management methods) from both the child’s and parents’ perspectives. This version did not have a chat feature.

Before its use in this study, the mobile app intervention BuddyCare underwent pilot-testing with 5 families at the Day Surgical Department of the children’s and adolescents’ clinic from December 2016 to January 2017. The purpose of the piloting phase was to test the usability of the mobile app from the perspective of families with children. The families of the children were requested to reach out to the hospital in case of any issues with the app or the preparation material provided. These issues could be related to the material’s clarity or quality or any other important concern from their perspective. On the basis of the piloting, the children’s families were satisfied with their user experience. Furthermore, they reported that they were confident about the preparation method used.

#### App Use by Parents

When a surgery was confirmed, hospital staff added the child, surgery type, and schedule to the app. The app then automatically selected the material suitable to that child and family. Hospital staff could monitor the family’s use of the app and receive and accept completed forms through the app. The app also reminded the family of how to tell the child about the surgery and offered other preparation instructions essential to the operation. The mobile app contained all the necessary information about the child’s surgery and made it possible to complete preinformation forms. It enabled parents to access information at convenient times whether they were at home, at work, or traveling or during their free time. Parents also had the option of getting in touch with the hospital if problems arose with the app.

Parents randomized to the IG received instructions for downloading the free BuddyCare app (Android or iOS) and its activation code 3 to 4 weeks before their child’s surgery. Alternatively, parents could access a web-based application portal via a web browser. The app reminded users about important instructions to be followed 2 weeks before and 1 week after the surgery. Hospital staff assigned a start date, time limit, and expiration date for various tasks and instructions based on their assessment of when particular information was relevant to the child and their family. The timeline used color coding and spacers to make it easy for both children and family members to comprehend. The family was also able to access information in different formats based on their preferences. All the children and parents undergoing the same surgery received the same information at the same time. Forms filled out through the app went directly to the hospital staff. Hospital staff could monitor the use rate of the app throughout the study, but its contents were not modified during the study. No problems with the app were reported during the study period. Families who did not use the app were contacted directly. In this study, we used the Template for Intervention Description and Replication (TIDieR) checklist and guide to ensure a clear description of the intervention implemented [47].

### CG Procedure

Parents in the CG received the conventionally established preparation for pediatric day surgery at the hospital. They received written preparation instructions before the procedure, which included information about the time of the surgery, what the planned surgery entailed, and recovery. In addition to the written instructions, the child was shown a video called “Juuso ja unikorkki” (“a boy named Juuso with an intravenous cannula ‘sleep cap’ in his hand”), after which the parents were able to contact the hospital with any further questions.

In addition, a nurse called the parents the day before the surgery to confirm the time of the operation, check on the child’s current health, complete a preinformation form for the child covering issues such as allergies and primary diseases, answer the parents’ questions, and give instructions on nutrition and arrival at the hospital. The content of this call was the same as that provided in the mobile app intervention. In this study, the implementation of the CG preparation was outlined in accordance with the TIDieR checklist [48].

### Assessments for Parents’ Anxiety and Stress and Children’s Pain and Fear

#### STAI (Parents)

The STAI was developed by Spielberger et al [49]. This self-reported anxiety instrument for parents includes 40 statements, of which 20 assess trait anxiety and 20 measure state anxiety (S-Anxiety). The S-Anxiety scale evaluates how the respondents feel at the time of taking the test, whereas the trait anxiety scale assesses a person’s general level of anxiety. This study only used the STAI S-Anxiety scale. Responses on the S-Anxiety scale assess the intensity of feelings “at this moment”: (1) *not at all*, (2) *somewhat*, (3) *moderately so*, and (4) *very much so*. There are 20 items, of which 10 are scored from 1 to 4, with 4 representing the highest level of anxiety, and the other 10 are scored the opposite way. We reversed the latter scores (4 became 1, 3 became 2, 2 became 3, and 1 became 4) so that, when evaluating the total score, a higher score represented a higher level of anxiety. The total score on the S-Anxiety scale ranges from mild (20-39) and moderate (40-59) anxiety to intense anxiety (60-80) [49]. The internal consistency of the pretranslated version of the questionnaire used in this study (Mind Garden) has been assessed. The internal consistency coefficients ranged from 0.86 to 0.95, whereas test-retest reliability estimates ranged from 0.65 to 0.75 over a 2-month interval [49]. According to Gustafson et al [50], the STAI demonstrates good reliability based on a Cronbach α of .93.

#### The Verbal Rating Scale for Stress (Parents)

The Verbal Rating Scale for Stress (VRSS) describes recent experiences of stress using the following scale: (0) *I did not feel any stress*, (1) *I felt a little stress*, (2) *I felt stress*, (3) *I felt quite a lot of stress*, (4) *I felt a lot of stress*, and (5) *I felt the worst stress I could think of*. Parents responded to the 6 claims comprising the VRSS on 4 distinct occasions. The VRSS has demonstrated appropriate reliability and validity in previous studies [51].

#### The Visual Analog Scale (Parents)

The visual analog scale (VAS) is a continuous, self-reported scale that measures a characteristic or attitude that is believed to vary over a given continuum (0-10). The scale is reliable and valid [52]. In this study, parents’ appraisal of their children’s pain was measured using the VAS (ie, a 100-mm horizontal line where 0=the child has no pain and 10=the child has the worst possible pain). We offered the following instruction to the parents: “As a parent, evaluate your child’s amount of pain and mark it in the section where it best explains your child’s pain (no pain—worst possible pain).” These measurements were taken 3 times a day from the day of the surgery for up to 3 days. All the responses were detailed on a single A4 leaflet daily. There was one leaflet for the children and one for the parents.

#### The Wong-Baker Faces Pain Rating Scale (Child)

The Wong-Baker Faces Pain Rating Scale (WBS) is one of the various face scales used in pediatric settings for pain estimation. Face scales are ordinal outcome measures that consist of a limited number of categorical responses following a specific design and are used primarily to describe the amount of pain in pediatric patients. Face scales are valid and reliable tools to measure procedural pain intensity [53,54]. The WBS has been widely used to obtain self-reported pain in children as young as 3 years of age and to rate pain harshness. The WBS is an exceptional measure of treatment effectiveness in school-age children and adolescents [53,55]. In this study, the parent told the child that each face represented a person who had either no pain (hurt), some pain, or a lot of pain; more specifically, face 0=*does not hurt at all*, face 2=*hurts just a little bit*, face 4=*hurts a little bit more*, face 6=*hurts even more*, face 8=*hurts a whole lot*, and face 10=*hurts as much as you can imagine*. The parent then asked the child to select the face that best represented the pain they were experiencing [53,55].

#### The Facial Affective Scale (Child)

The Facial Affective Scale (FAS) is a bipolar 9-point measure that includes a child’s emotional (affective) reaction to pain, ranging from “happiest feeling possible” to “saddest feeling possible.” The FAS was used once at home 3 days after the surgery. Parents were told the following: “the facial images indicate the child’s emotions, and the child should choose the face that best expresses their state of fear.” Once the child had chosen, the parent should circle it. The reliability and validity of the FAS have been established [56,57].

#### The Parents’ Postoperative Pain Measure (Parent)

The Parents’ Postoperative Pain Measure (PPPM) was developed in Canada to help parents assess their children’s postoperative pain. It consists of 15 items describing behavior changes in children aged 2 to 6 years after they are discharged from the hospital following day surgery [58]. The PPPM comprises a series of *yes or no* questions such as the following: “is your child whining or complaining more than usual?” The PPPM findings are valid and reliable for measuring Finnish children’s postoperative pain [59]. Parents completed the PPPM for all children in this study at home 3 days after surgery.

### Data Collection

A pediatric surgeon assessed the need for surgery and placed the child in the surgery queue. After planning the surgery, the schedule went to secretaries who had been trained by the researcher to facilitate the study. Patients suitable for the study were selected from the schedule according to the selection criteria. Each month, the secretaries provided the researcher with information on the number of study families identified and their details. The researcher then called the families and asked about their willingness to participate in the study. After receiving oral consent, the secretaries sent the families written informed consent forms related to the study, instructions, and questionnaires. The parents were informed about the study both verbally and in writing. Families in the IG also received instructions for logging in to the mobile app and using it, through which they could access instructions for preparing for the surgery. The CG received the conventional preparation instructions.

On arrival at the hospital, parents brought the written consent form along with the first-stage assessment (T1), which they had completed at home. This first baseline assessment included general demographic data along with parents’ self-reported stress and anxiety and children’s reported pain and fear. After the physician met the child and made the final decision for surgery, a nurse collected follow-up data at the hospital before the child’s procedure (T2), after the surgery, and before the child was discharged from the hospital (T3). In the second (T2) and third (T3) measurements, parents were asked to rate the stress they experienced. After the child and parents returned home, one parent took the study-related measures for 3 days at home, 1 to 3 times a day depending on the assessment (T4). In the second assessment, parents self-reported their anxiety and stress and their child’s self-reported pain and fear regarding care (Figure 2). The forms sent home to parents were coded using the same code that was used for T2 and T3. The questionnaires designed for parents with children were organized in a manner such that each page featured only 1 measure and provided concise instructions for completing it.

This study was guided by the CONSORT (Consolidated Standards of Reporting Trials) checklist [60] (Figure 1) and registered at ClinicalTrials.gov (NCT03774303).

### Ethical Considerations

This study received ethics approval from the Northern Ostrobothnia Regional Ethics Committee Board (EETTMK:53/2017) in June 2017. This study was conducted in accordance with the Declaration of Helsinki. Ethical considerations were respected at all stages of the study, including the voluntary participation of family members, the right to information about the research before and throughout data collection, the right to ask questions, the right to be treated with respect and honesty, and the right to suspend research. Participants’ privacy and data protection were ensured throughout the data collection process [61].

The parents were informed about the study both orally and in writing. Before the study began, the researcher sent a notice to the enrolled parents. The researcher then approached them for written informed indication of their willingness to participate in the investigation [61]. The questionnaires collected the child’s family name and year of birth without their personal identification number. A register was then created to anonymize the participants during the data collection process. The researcher was responsible for the privacy of the materials collected and followed ethical principles to ensure that others could not access the materials. No public metadata have been made available based on the data gathered for this study.

### Data Analysis

Descriptive statistics (frequencies with percentages, medians with IQRs, and means with SDs) were used to express the parents’ characteristics and study variables. Normality of the continuous variables was first inspected visually using histograms separately for the IG and CG and confirmed using the Shapiro-Wilk test of normality. The S-Anxiety sum score was found to be normally distributed, whereas the child’s pain, assessed by parents, nurses, and the child, and the child’s fear were skewed in at least some of the assessments. Parents’ stress was categorical. Parents’ stress was further divided into 3 categories: no stress (VRSS=0), mild stress (VRSS=1), and moderate to intense stress (VRSS=2-5). The child’s pain, assessed by the child, was divided into 3 categories: no pain (WBS=0), moderate pain (WBS=2-4), and severe pain (WBS=6-10). An intention-to-treat analysis was performed, which meant that all parents with children participating in the study were analyzed in the groups to which they were initially randomized.

The significance of between-group differences in the variables measured was assessed using the 2-tailed *t* test (S-Anxiety sum score), chi-square test (categorized study variables), or Mann-Whitney *U* test (child’s pain, assessed by parents and nurses [VAS], and child’s fear [FAS]). Changes between each assessment were analyzed separately for the IG and CG using paired-sample 2-tailed *t* tests (S-Anxiety sum score), sign test (categorized study variables), or Wilcoxon signed rank test (child’s pain, assessed by parents, and child’s fear) with Benjamini-Hochberg correction and a false positive rate of 0.05 for multiple comparisons. In the analyses of change, data from those who replied to all assessments were used (T1, T2, T3, and T4). The pertinent effect sizes for all comparisons were calculated and are explained in detail in Multimedia Appendix 1. In addition, the change in the S-Anxiety sum score was analyzed using a linear mixed model with assessment (T1 and T4) as a repeated factor and intervention and gender as fixed factors. The threshold for statistical significance was set at a *P* value of ≤.05. The analyses were performed using the SPSS statistical software for Windows (version 28; IBM Corp).

### Validity, Reliability, and Rigor

Randomization was performed after each participant provided informed consent following telephone contact with the researcher. Parents were informed about the study both verbally and in writing. Intention-to-treat analysis was used as the analyses were to be performed in the original randomization groups and to maintain comparability between groups, reduce the effects of bias, and highlight the effects of the intervention used [62,63]. To ensure the fidelity of the intervention, the researcher provided training to all the nurses who assisted with the research. This training covered the purpose and design of the trial, data collection procedures, and ethical requirements. Employees from Buddy Healthcare trained the hospital staff on using the mobile app intervention. The reporting of the study results conforms to the CONSORT statement [60]. The TIDieR checklist improved the reporting of the intervention [64]. All data were collected from parents or preschool children using standardized data collection forms (ie, STAI, VRSS, WBS, FAS, PPPM, or VAS). The effect sizes calculated for the variables strengthened the reliability of the results (Multimedia Appendix 1).

## Results

### Characteristics of the Research Participants

A total of 70 children and their parents were recruited for this study. The IG comprised 51% (36/70) of the participants (loss rate: 8/36, 22%), and the CG comprised 49% (34/70; loss rate: 8/34, 24%) of the participants. A total of 54 responses from participants in the IG (28/36, 78%) and CG (26/34, 76%) were analyzed (participation rate of 54/70, 77%; Figure 2). No significant differences were observed in the background data about parents in the 2 groups apart from gender distribution (*P*=.004)—the CG comprised 27% (7/26) of men, whereas the IG included no men (Table 1).

**Table 1:** Comparison of baseline demographic information between the intervention and control groups (n=54).

			Intervention group (n=28), n (%)		Control group (n=26), n (%)		Total, n (%)		*P* value	
Participants			28 (100)		26 (100)		54 (100)		—^a^	
**Parent age category (years)**										.08
	25-30	8 (29)		6 (23)		14 (26)				
	31-35	6 (21)		6 (23)		12 (22)				
	36-40	10 (36)		10 (38)		20 (37)				
	41-45	2 (7)		4 (15)		6 (11)				
	46-50	1 (4)		0 (0)		1 (2)				
	>50	1 (4)		0 (0)		1 (2)				
**Sex**										.004
	Female	28 (100)		19 (73)		47 (87)				
	Male	0 (0)		7 (27)		7 (13)				
**Marital status**										>.99
	Married	21 (75)		20 (77)		41 (76)				
	Cohabitation	5 (18)		6 (23)		11 (20)				
	Single parent	1 (4)		0 (0)		1 (2)				
	Other	1 (4)		0 (0)		1 (2)				
**Educational level**										.55
	No education	1 (4)		2 (8)		3 (6)				
	Vocational education	15 (54)		9 (35)		24 (44)				
	College or polytechnic education	7 (25)		9 (35)		16 (30)				
	University education	5 (18)		6 (23)		11 (20)				
**Child’s age (years)**										.69
	2	4 (14)		1 (4)		5 (9)				
	3	2 (7)		4 (15)		6 (11)				
	4	7 (25)		6 (23)		13 (24)				
	5	8 (29)		8 (31)		16 (30)				
	6	7 (25)		7 (27)		14 (26)				
**Previous hospital experience**										.65
	No	10 (36)		13 (50)		23 (43)				
	Yes, once	10 (36)		7 (27)		17 (31)				
	Yes, many times	8 (29)		6 (23)		14 (26)				
**Previous hospital experience quality (n=31)**			18 (58)		14 (45)		31 (100)		.78	
	Good	14 (78)		13 (93)		26 (84)				
	Quite good	3 (17)		1 (7)		4 (13)				
	Poor	1 (6)		0 (0)		1 (3)				
**Child regular pain medication**										—
	No	28 (100)		26 (100)		54 (100)				
	Yes	0 (0)		0 (0)		0 (0)				

^a^*P* values were only calculated as a whole between the intervention group and the control group to describe the differences in the age and sex of the groups. Therefore, not all columns will have numerical values.

### Parents’ Anxiety

The anxiety experienced preoperatively at home and postoperatively at home did not differ between the IG and CG. The mean for preoperative anxiety was 36.7 (SD 9.9; 28/36, 78%) in the IG and 36.9 (SD 12.3; 26/34, 76%) in the CG (*P*=.95). The mean postoperative anxiety was 28.1 (SD 6.9; 24/36, 67%) in the IG and 30.2 (SD 7.06; 17/34, 50%) in the CG (*P*=.34; Multimedia Appendix 2).

Preoperatively, parents in both groups experienced mild to moderate anxiety. Postoperatively, most parents in both groups experienced mild anxiety. Parental anxiety did not differ between the groups preoperatively (mild: IG=19/28, 68% and CG=18/26, 69%; moderate: IG=9/28, 32% and CG=7/26, 27%; intense: IG=0% and CG=1/26, 4%; *P*=.77) or postoperatively (mild: IG=21/24, 88% and CG=16/17, 94%; moderate: IG=3/24, 13% and CG=1/17, 6%; intense: IG=0% and CG=0%; *P*=.63; Multimedia Appendices 2 and 3).

According to the results, anxiety decreased significantly in both groups (IG: *P*=.003; CG: *P*=.002) when comparing measurements before and after surgery. The relevant data are presented in Multimedia Appendix 4. On the basis of the linear mixed model, there was no difference between the IG and CG (*P*=.13), but female individuals experienced more anxiety than male individuals (*P*=.02), and overall anxiety decreased significantly (*P*<.001).

**Figure 3:**
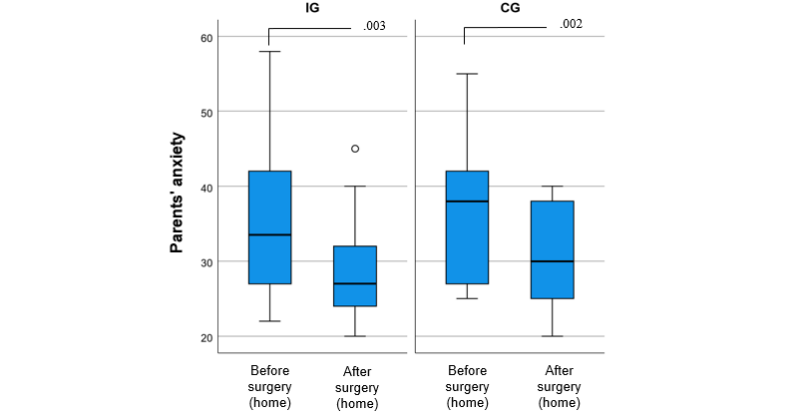
Parents’ pre- and postoperative anxiety levels in the intervention group (IG; n=22) and control group (CG; n=17), with *P* values of <.05 between the assessments.

Overall, parental anxiety decreased from the preoperative assessment (mean 36.3, SD 10.3) to the postoperative assessment (mean 29.4, SD 6.9), the average change being −7.0 (95% Cl −9.8 to −4.1; *P*<.001). Separately, within-group changes were −7.0 (95% CI −11.3 to −2.6) in the IG and −7.0 (95% CI −11.0 to −3.0) in the CG (Figure 3).

### Parents’ Stress

**Figure 4:**
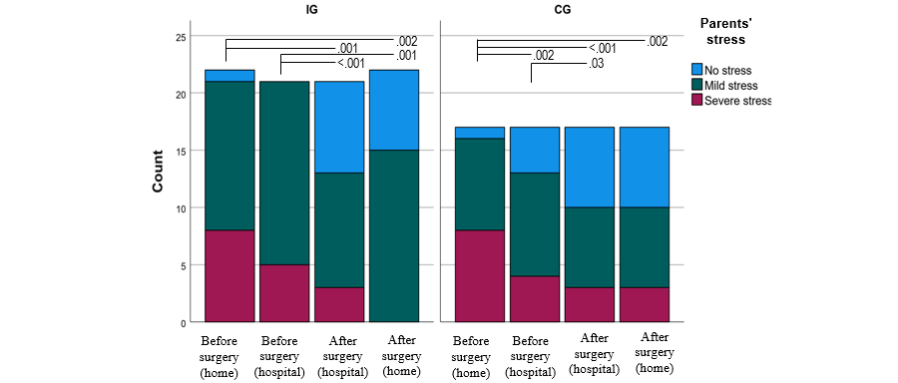
Parents’ pre- and postoperative stress levels in the intervention group (IG; T1: n=28; T2: n=23; T3: n=32; T4: n=24) and control group (CG; T1: n=26; T2: n=26; T3: n=26; T4: n=17), with *P* values of <.05 between the assessments.

The amount of stress experienced at home by parents in both groups was very similar before surgery. Preoperatively at home, most parents in both groups experienced no stress or mild stress (17/28, 61% in the IG and 13/26, 50% in the CG; *P*=.61). Preoperatively at the hospital, most of the parents in the IG felt mild (24/31, 77%) or moderate to intense (7/31, 23%) stress, whereas in the CG, 23% (6/26) of the parents felt no stress. The difference between the groups was statistically significant (*P*=.02; Multimedia Appendix 2). Postoperatively at the hospital, most parents in both groups experienced no stress (15/32, 47% in the IG and 13/26, 50% in the CG; *P*>.99). Postoperatively at home, none of the parents in the IG and 18% (3/17) in the CG experienced moderate to intense stress. The difference between the groups was statistically significant (*P*=.05; Multimedia Appendix 3). The amount of stress decreased significantly from before to after surgery in both groups (from T1 to T4: *P*=.003 in the IG and *P*=.004 in the CG; Figure 4).

### Children’s Pain

Parents’ evaluations of their children’s pain did not differ between the IG and CG. Almost all parents rated the child’s pain preoperatively at a maximum of 1 on the VAS at home (IG: 22/25, 88%; CG: 23/26, 88%) and at the hospital (IG: 28/31, 90%; CG: 22/26, 85%). Postoperatively at the hospital, 52% (16/31) of parents in the IG and 35% (9/26) of parents in the CG rated the child’s pain on the VAS at between 1.1 and 3.0, and 13% (4/31) of parents in the IG and 31% (8/26) of parents in the CG rated it as >3. Postoperatively at home, 25% (5/20) of parents in the IG and 40% (6/15) of parents in the CG rated their child’s pain on the VAS at between 1.1 and 3.0, and 30% (6/20) of parents in the IG and 20% (3/15) of parents in the CG rated it at >3. The median VAS score was 0.1 (IQR 0.0-0.4) in the IG and 0.0 (IQR 0.0-0.3) in the CG preoperatively at home (*P*=.25) and 0.0 (IQR 0.0-0.2) in the IG and 0.0 (IQR 0.0-0.2) in the CG preoperatively at the hospital (*P*=.98). The median VAS score was 1.4 (IQR 0.5-3.4) in the IG and 1.5 (IQR 0.5-2.3) in the CG (*P*=.84) postoperatively at the hospital and 1.8 (IQR 0.4-2.4) in the IG and 1.6 (IQR 0.2-3.6) in the CG (*P*=.72) postoperatively at home. The parents’ assessments increased in both groups from home to back home, but this was only statistically significant in the CG (from T1 to T4: *P*=.06 in the IG and *P*<.001 in the CG; Multimedia Appendices 2 and 3).

**Figure 5:**
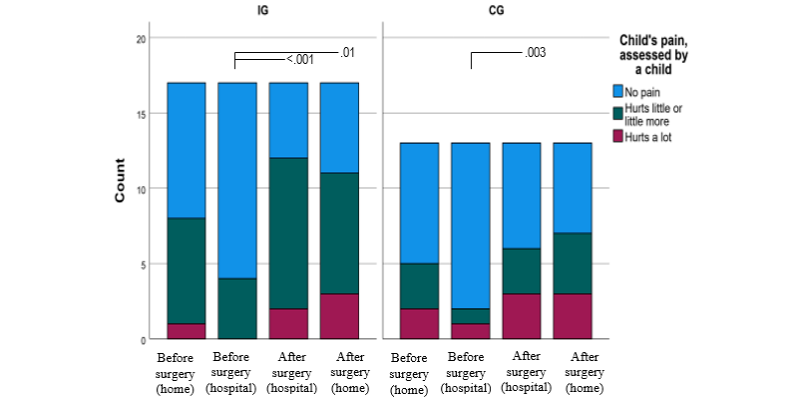
Children’s pre- and postoperative pain assessed in the intervention group (IG; T1: n=23; T2: n=32; T3: n=28; T4: n=20) and control group (CG; T1: n=26; T2: n=26; T3: n=26; T4: n=13), with *P* values of <.05 between the assessments.

The children in both the IG and CG experienced pain similarly according to their own assessment (Figure 5). Preoperatively at home, 61% (14/23) of the children in the IG and 73% (19/26) of the children in the CG reported feeling no pain, and 4% (1/23) of the children in the IG and 12% (3/26) of the children in the CG reported severe pain. Preoperatively at the hospital, 82% (23/28) of the children in the IG and 89% (23/26) of the children in the CG reported feeling no pain, and none of the children in the IG and 8% (2/26) of the children in the CG reported severe pain. The rest reported experiencing moderate pain (Multimedia Appendix 2). Postoperatively at the hospital, 40% (12/28) of the children in the IG and 50% (12/26) of the children in the CG reported feeling no pain, and 13% (4/28) of the children in the IG and 21% (5/26) of the children in the CG reported severe pain. Moderate pain was reported by 47% (14/28) of the children in the IG and 29% (7/26) of the children in the CG (Multimedia Appendix 3). Postoperatively at home, 40% (8/20) of the children in the IG and 46% (6/13) of the children in the CG reported feeling no pain, and 15% (3/20) of the children in the IG and 23% (3/13) of the children in the CG reported severe pain (Multimedia Appendix 3).

From T1 to T4, parents rated a decrease in pain for 19% (3/16) of the children in the IG and 7% (1/15) of the children in the CG and an increase in pain in 75% (12/16) of the children in the IG and 73% (11/15) of the children in the CG. According to the children’s ratings, pain decreased for 29% (5/17) of the children in the IG and 15% (2/13) of the children in the CG and increased for 47% (8/17) of the children in the IG and 46% (6/13) of the children in the CG.

Nurses’ evaluations of the children’s pain at the hospital did not differ between the IG and CG. Almost all nurses rated the children’s pain preoperatively at a maximum of 1 on the VAS (IG: 31/32, 97%; CG: 25/26, 96%), and postoperatively, nurses rated the pain of 25% (8/32) of the children in the IG and 31% (8/26) of the children in the CG on the VAS at between 1.1 and 3.0 and of 9% (3/32) of the children in the IG and 23% (6/26) of the children in the CG as >3. The median VAS score was 0.0 (IQR 0.0-0.2) in the IG and 0.0 (IQR 0.0-0.5) in the CG preoperatively (*P*=.81) and 0.7 (IQR 0.0-1.9) in the IG and 1.3 (IQR 0.0-3.1) in the CG postoperatively (*P*=.54). The nurses’ assessments increased in both groups from preoperative assessment to postoperative assessment and were similar to the parents’ assessments of pain (Multimedia Appendices 2 and 3).

### Children’s Fear

**Figure 6:**
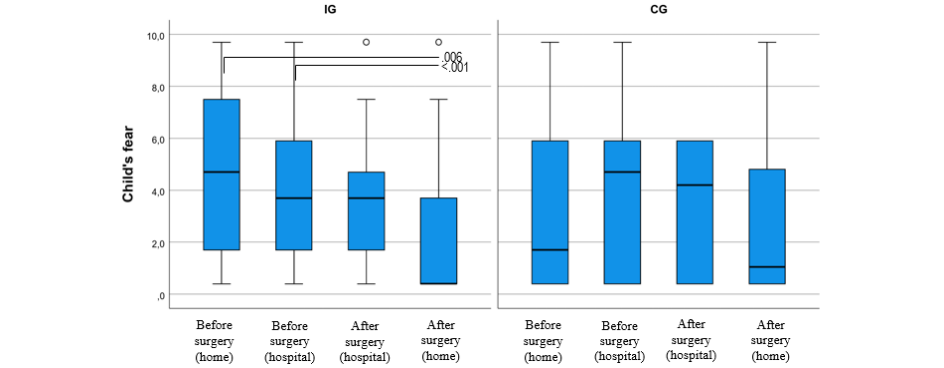
Children’s pre- and postoperative fear assessed in the intervention and control groups, with *P* values of <.05 between the assessments. CG: control group; IG: intervention group.

The median FAS did not differ between the IG and CG preoperatively (at home: median 4.7, IQR 1.4-7.6 for the IG and median 2.7, IQR 0.4-7.5 for the CG with *P*=.20; at the hospital: median 3.7, IQR 1.7-5.9 for the IG and median 4.2, IQR 0.4-4.2 for the CG with *P*=.59) or postoperatively (at the hospital: median 3.7, IQR 0.4-4.7 for the IG and median 3.7, IQR 1.1-5.9 for the CG with *P*=.62; at home: median 0.4, IQR 0.4-4.0 for the IG and median 1.1, IQR 0.4-5.4 for the CG with *P*=.81; Multimedia Appendices 2 and 3; Figure 6). Fear decreased from T1 to T4 in 65% (11/17) of the children in the IG and 44% (7/16) of the children in the CG and increased in 18% (3/17) of the children in the IG and 31% (5/16) of the children in the CG. Fear decreased in both groups, but this change was statistically significant only in the IG (*P*=.006 in the IG and *P*=.44 in the CG; Multimedia Appendix 4; Figure 6).

### Child Postoperative Pain

The median postoperative pain did not differ between the IG and CG (1.0, IQR 0.0-2.8 for the IG and 1.0, IQR 0.0-3.5 for the CG; *P*=.36).

### Summary of Results

This randomized controlled trial provides evidence that the tested mobile app performs comparably with the established methods for preparing preschool children and their parents for day surgery.

## Discussion

### Principal Findings

This study aimed to evaluate the effectiveness of a mobile app intervention in preparing the parents of preschool children for day surgery. The mobile app intervention did not reduce anxiety or pain levels. Both the IG and CG experienced a significant decrease in their anxiety levels from before to after the surgery. Before undergoing surgery at the hospital, most parents in the IG (24/31, 77%) reported experiencing mild stress. In contrast, in the CG, only 23% (6/26) of parents reported feeling no stress. This discrepancy between the 2 groups was deemed statistically significant. After the surgery, parents in the IG experienced less stress compared with those in the CG, and the difference was significant. In addition, both groups showed a decrease in stress levels from before to after the surgery within their respective groups. In addition, fear levels in children in the IG decreased over time, whereas no significant change was observed in those in the CG. The level of fear experienced by the participants decreased after the surgery within their respective groups. However, statistical analysis indicated that this decrease was only significant in the IG and not in the CG. It is worth noting that a mobile app may be a cost-effective alternative to established methods for providing parents with the support they need. This creates a positive rationale for the future use of the mobile app that we tested.

### Comparison With Prior Work

The results showed that the parents in the IG experienced more stress before the surgery at the hospital as more parents in the CG were not stressed at all (2/6, 33% female and 4/6, 67% male). According to Lööf and Lönnqvist [27], parents’ background factors, such as gender, age, and cognitive abilities, affect the stress they experience. Typically, mothers experience more stress than fathers [23,36]. Parents may experience a rise in stress when transitioning from home to the hospital during a procedure because of a lack of previous interaction with hospital staff. Studies suggest that effective preparation for day surgery involves not only providing more information but also promoting seamless collaboration and social support [5,65,66] as well as the ability to ask questions and receive clear instructions [67,68]. However, after the event concluded, the stress levels of parents in both groups showed a statistically significant decrease according to their perception.

In this study, single-parent families may have experienced more stress before and after surgery, possibly because of a lack of support from another adult. However, the difference compared with unmarried or married parents was not statistically significant and requires further analysis. According to Cagiran et al [69], enabling communication with HCPs and allowing parents to talk to other parents in similar situations can reduce anxiety and help parents support their children more effectively. It is essential to consider this dynamic when designing new preparation methods. It is also important to weigh whether these social interactions should occur through face-to-face or digital channels (eg, Teams [Microsoft Corporation] or Zoom [Zoom Video Communications]). We already know that distinct advantages of mobile health (mHealth) apps are the sheer amount of text and visual content that they can offer and the various reminders that are built into mobile apps that can support, guide, and monitor families’ preparation as well as address any problems that may arise [39]. Furthermore, allowing families to engage in the preparation process from the comfort of their own homes may make them more receptive and able to retain procedure-related information and skills training.

On the basis of the findings presented, it is common for parents to experience mild anxiety and stress before their child’s day surgery, which is consistent with a study conducted by Justus et al [31]. Notably, in this study, parents in the CG experienced more postoperative stress at home than at the hospital compared with those in the IG. The stress levels of parents in the CG did not decrease significantly compared with anxiety after their child’s surgery. This could be due to their concerns about their child’s recovery, pain management, and potential nausea. In contrast, parents in the IG felt more secure as they continued to use the mobile app at home after the surgery. Previous studies indicate that parents want to support their children in the best possible way [5,6], which requires clear guidance, support, and instructions from HCPs during the postoperative period. It is essential to avoid transmitting parental anxiety and stress to the child as it can cause fear and pain, ultimately slowing down the child’s postoperative recovery [32]. Therefore, HCPs should focus on providing continuity of care after the surgery. With the increasing number of day surgeries among preschool children worldwide, exploring this topic in future research is vital. The mobile app could also be enhanced by providing more diverse and up-to-date information about the care unit, internet-based features such as games and virtual reality environments, and a chat service for users. According to Armfield et al [70], software-based video tools can effectively deliver telemedicine services and are feasible.

According to our study, slightly fewer than half (23/48, 48%) of the children in both the IG and CG experienced fear or mild fear at home before the surgery. This broadly corresponds with the findings of Gates et al [29], which were that up to 30% of children experience moderate or significant pain related to a day surgery. Parents’ participation in a preschool child’s day surgery increases the sense of security for both the child and the parents, enables family togetherness, reduces children’s pain and fear, and lowers parents’ anxiety and stress [71,72]. It is worth noting that the level of fear expressed by children in the IG decreased more following the surgery than that of children in the CG, where such a drastic change did not occur. According to Salmela et al [73], parents’ involvement and user-friendly preparation support a child’s survival strategies. Positive mental images can also support them and influence their sense of control over the situation they are facing [73]. Our research indicates that the mobile app we tested could be effective in both preparing the child and supporting their recovery. Furthermore, our finding that children in the IG experienced less fear shows that drug-free methods can influence not only preoperative pain [74] but also fear in the postoperative phase. According to Wennström and Bergh [75], distinguishing between pain and fear can be difficult for children. However, in this study, the children were well able to distinguish between the fear and the pain that they experienced.

On the basis of this study, the children’s experiences of pain and fear did not align. The findings showed that children in both groups had higher levels of fear than pain before the surgery. Postoperatively, both groups experienced a rise in pain but a decrease in fear, although the decrease was slightly less noticeable in the CG.

On the basis of the results presented, using a mobile app is as effective as the established preparation method. Several previous studies [23,76] have reported that parents experience significant stress and anxiety during their child’s hospitalization. However, our research shows that this need not be the case. The levels of anxiety and stress experienced by parents can be affected by high-quality and sufficiently adaptable preparation. To reduce parental anxiety, it is essential to provide accurate information before and during a child’s hospital stay [10,69]. The main objective of preparing a family for pediatric day surgery should be to introduce them to the care path and lay out any issues that are essential for the trip from home to the hospital, as well as on returning home following discharge [37]. To ensure continuity of care, families need to be well informed about recovery from the surgery and how they can support it once the child is back home.

This study suggests that this can be achieved using a mobile app intervention that is designed to support the needs of both HCPs and families in a customer-oriented and adaptable way. Good methods can also increase families’ satisfaction with their children’s care [15,77]. mHealth apps may assist in day surgery preparation and simultaneously establish a positive interaction between patients and HCPs [78]. When used best, information and communications technology can increase the accessibility, equity, effectiveness, and quality of health promotion services, thus contributing to a more rational use of health services [79,80]. It is important to remember that the primary purpose of digitalization is not merely to transform the information from paper into an electronic format but also to take full advantage of the many digital tools available to users. At the same time, digitalization must leave adequate room for authentic social interaction.

An mHealth app enables relevant information and other preparation materials such as images and videos to be made immediately available to parents regardless of time, place, or context [78]. Well-designed mobile apps provide families with a broad range of information that is presented in different ways. This makes them useful and appropriate for diverse families of different backgrounds and in different circumstances (eg, frequent travelers or those who need more specific guidance). Every individual has distinct preferences, and Kampouroglou et al [81] have shown that certain parents prefer images and videos over written information. Furthermore, McCloskey et al [82] and Tozzi et al [83] have argued that expanding the use of mobile apps to preschool children is justified as building skills early also supports their later development, particularly when it takes place with parents.

According to this study, single parents or those reported as “others” experienced more stress before and after the surgery as compared with 2-parent families. When preparing for day surgery, it should be noted that not all families necessarily have an extensive social network. According to Winkler et al [84], parents also need support, information, and advice on how to prepare and support their child and manage their own emotions. According to Cagiran et al [69], communication with health professionals or other parents in the same situation reduces anxiety and helps parents support their children. The development of any preparation method must consider diverse family structures and how different families are supported by the community around them [42]. This matters as it is difficult to reduce parental anxiety if parents cannot present their questions and concerns about their child’s operation [5]. Previous research has shown that certain types of parents benefit from interactive guidance along with other methods [38]. It is also important that HCPs identify and inform themselves about families who have had negative previous experiences of hospital visits as such incidents affect the level of anxiety experienced by parents. Parents should also be afforded the opportunity to contact HCPs in the postoperative period as this allows them to seek advice about their child’s care and potentially receive support if there are symptoms of acute stress disorder following the surgery [85]. This type of communication should always be reciprocal [44]. Agbayani et al [14] have noted that more evidence-based information is needed on digital solutions for reducing parents’ anxiety and stress.

This study provides a fresh perspective for HCPs, showing that a mobile app can adequately prepare preschool children for day surgery. This confirms that users already perceive digital preparation methods as acceptable and feasible. However, there is a need for more up-to-date research on family experiences of pediatric surgery so that newly developed interventions can provide families with the care and support they truly need.

### Limitations

Although this study was designed to maximize validity and reliability, it has certain limitations. The sample size was initially increased to account for any potential dropouts, but it was not large enough to detect significant between-group differences as some parents did not answer some of the questions. The reliability of the results can also be affected by the statistical difference between the groups (*P*=.004) regarding the gender of the parents. Women experienced more stress than men before the operation, so especially the results concerning stress should be interpreted with caution. Some parents withdrew from participating partway through the study because of their desire to monitor their child’s ailment and concern that surgery may harm their child permanently. The resulting missing values could not be substituted with mean values because of the small sample size. Attempts were made to improve response rates by sending families 2 reminder messages, one 4 days after surgery and the other 14 days after surgery. The study design also made blinding difficult. It was impossible to blind the nurses as they needed to know which parents were in the CG so that they could call them the day before the surgery. In addition, the nurses needed to know which families were using the mobile app so that they could track their progress. This solution was chosen as a research nurse was not available. However, both groups were deliberately provided with the same guidance material for the child’s surgery; only the delivery method differed. Parents in the IG received all the information via a mobile app, whereas parents in the CG received information in writing, through a video (*Juuso ja unikorkki*), and via a telephone call from the nurse. It should be stated that a few families had previous hospital experiences, which may have biased their perceptions of stress and anxiety before, during, and after surgery. Study parts 1 and 4 were conducted at the parents’ homes, with the respondents answering the questions themselves. The study used a self-reported outcome assessment, which may have caused social bias. To increase transparency, all the study results concerning the participating parents, including statistically insignificant results, have been reported. With regard to participants, all the parents in this study were recruited from one university hospital, which may reduce the generalizability of the results. In the future, the effectiveness of a mobile intervention should be assessed over a longer study period.

### Conclusions

The mobile app intervention did not reduce anxiety or pain levels. However, it was observed that parents in the IG experienced statistically significant heightened stress levels before surgery at the hospital, which decreased significantly after the surgery at home. In addition, fear levels in children in the IG decreased over time, whereas no significant change was observed in the CG. In the future, mobile app interventions can be used instead of traditional approaches for preparing preschool children and their parents for day surgery without increasing the psychological burden. However, a mobile app cannot completely replace face-to-face contact, and the results presented about the anxiety experienced by single parents highlight the need to consider interactions between parents and HCPs. Hence, future developments will need to consider the individual characteristics and needs of families to ensure sufficient family-based preparation. This will be essential to develop health care value chains further and offer new effective and customer-driven solutions for families involved in pediatric care.

## Appendix

Multimedia Appendix 1 Effect sizes of the research results.

Multimedia Appendix 2 Comparison of parents’ anxiety and stress and children’s pain and fear between the intervention and control groups in the assessments before the day surgery.

Multimedia Appendix 3 Comparison of parents’ anxiety and stress and children’s pain and fear between the intervention and control groups in the assessments after the day surgery.

Multimedia Appendix 4 Comparison of parents’ anxiety and stress and children’s pain and fear from before to after the day surgery in the assessments at home.

Multimedia Appendix 5 CONSORT-EHEALTH (V 1.6.1) - Submission/Publication Form.

## Abbreviations

CG: control group
CONSORT: Consolidated Standards of Reporting Trials
FAS: Facial Affective Scale
HCP: health care professional
IG: intervention group
mHealth: mobile health
PPPM: Parents’ Postoperative Pain Measure
S-Anxiety: state anxiety
STAI: State-Trait Anxiety Inventory
TIDieR: Template for Intervention Description and Replication
VAS: visual analog scale
VRSS: Verbal Rating Scale for Stress
WBS: Wong-Baker Faces Pain Rating Scale

## Data Availability

The data sets generated and analyzed during this study are available from the corresponding author upon reasonable request.
