# Supplementary material for: Effectiveness of a Mobile App Intervention for Preparing Preschool Children and Parents for Day Surgery: Randomized Controlled Trial
Source: J Med Internet Res. 2023 Sep 29;25:e46989. doi: 10.2196/46989 (PMC10576237; doi:10.2196/46989)
Supplement: Multimedia Appendix 1 [file jmir_v25i1e46989_app1.docx]

Appendix 1. Effect sizes of research results. Table S1.

|  | **Group comparisons at different timepoints** | | | | **Change from T1 to T4** | |
| --- | --- | --- | --- | --- | --- | --- |
|  | **T1 IG vs. CG** | **T2 IG vs. CG** | **T3 IG vs. CG** | **T4 IG vs. CG** | **IG, T1 vs. T4** | **CG, T1 vs. T4** |
| **Parent’s anxiety (STAI-S)** | 0.018^1^ | - | - | 0.301^1^ | 0.619^2^ | 0.831^2^ |
| **Parent’s anxiety categories** | 0.149^3^ | - | - | 0.110^3^ | 0.300^4^ | 0.500^4^ |
| **Parent’s stress (VRSS)** | 0.125^3^ | 0.375^3^ | 0.031^3^ | 0.387^3^ | 0.423^4^ | 0.500^4^ |
| **Child’s pain, evaluated by parent (VAS)** | 0.026^5^ | <0.001^5^ | 0.002^5^ | 0.001^5^ | 0.117^5^ | 0.297^5^ |
| **Child’s pain, evaluated by nurse (VAS)** | - | 0.001^5^ | 0.007^5^ | - | - | - |
| **Child’s pain, evaluated by child (WBS)** | 0.244^3^ | 0.292^3^ | 0.183^3^ | 0.152^3^ | 0.227^4^ | 0.375^4^ |
| **Child’s fear (FAS)** | 0.035^5^ | 0.006^5^ | 0.005^5^ | 0.002^5^ | 0.205^5^ | 0.021^5^ |

^1^ Hedge’s g: 0.20–<0.50 small, 0.50–<0.80 medium, ≥0.80 large

^2^ d_repeated measures_: 0.20–<0.50 small, 0.50–<0.80 medium, ≥0.80 large

^3^ Cramer’s V, k=2: 0.10–<0.30 small, 0.30–<0.50 medium, ≥0.50 large

^4^ Cohen’s g: 0.05–<0.15 small, 0.15–<0.25 medium, ≥0.25 large

^5^ eta squared: 0.01–<0.06 small, 0.06–<0.14 medium, ≥0.14 large

Hedge’s g, d_repeated measures_, and eta squared were calculated using the web-based calculator (https://www.psychometrica.de/effect_size.html) and Cramer’s V and Cohen’s g using R version 4.3.0, package rcompanion.
