# Supplementary material for: Effectiveness of a Mobile App Intervention for Preparing Preschool Children and Parents for Day Surgery: Randomized Controlled Trial
Source: J Med Internet Res. 2023 Sep 29;25:e46989. doi: 10.2196/46989 (PMC10576237; doi:10.2196/46989)
Supplement: Multimedia Appendix 2 [file jmir_v25i1e46989_app2.docx]

**Table S2.** Comparison of parent's anxiety, stress and children`s pain and fear between the IG and CG in **the assessments before the day surgery**.

|  | **At home** | | | | | **In hospital** | | | | |
| --- | --- | --- | --- | --- | --- | --- | --- | --- | --- | --- |
|  | N | | Group | |  |  | |  | |  |
|  | IG | CG | IG | CG | P | IG | CG | IG | CG | P |
| **Parent’s anxiety (STAI-S), mean (SD)^1^** | 28 | 26 | 36.7 (9.9) | 36.9 (12.3) | 0.946 | n.a. | n.a. |  |  |  |
| **Parent’s anxiety categories, n (%)^2^** |  |  |  |  | 0.770 | n.a. | n.a. |  |  |  |
| Mild (20–39) |  |  | 19 (67.9) | 18 (69.2) |  |  |  |  |  |  |
| Moderate (40–59) |  |  | 9 (32.1) | 7 (26.9) |  |  |  |  |  |  |
| Intense (60–80) |  |  | 0 (0.0) | 1 (3.8) |  |  |  |  |  |  |
| **Parent’s stress (VRSS), n (%)^2^** | 28 | 26 |  |  | 0.607 | 31 | 26 |  |  | 0.017 |
| No stress (0) |  |  | 1 (3.6) | 2 (7.7) |  |  |  | 0 (0.0) | 6 (23.1) |  |
| Mild stress (1) |  |  | 17 (60.7) | 13 (50.0) |  |  |  | 24 (77.4) | 15 (57.7) |  |
| Moderate to intense stress (2–5) |  |  | 10 (35.7) | 11 (42.3) |  |  |  | 7 (22.6) | 5 (19.2) |  |
| **Child’s pain, evaluated by parent (VAS), median (IQR)^3^** | 25 | 26 | 0.1 (0.0–0.4) | 0.0 (0.0–0.3) | 0.254 | 31 | 26 | 0.0 (0.0–0.2) | 0.0 (0.0–0.2) | 0.983 |
| **Child’s pain, evaluated by nurse (VAS), median (IQR)^3^** | n.a. | n.a. |  |  |  | 32 | 26 | 0.0 (0.0–0.2) | 0.0 (0.0–0.05) | 0.809 |
| **Child’s pain, evaluated by child (WBS), n (%)^2^** | 23 | 26 |  |  | 0.243 | 28 | 26 |  |  | 0.107 |
| No pain (0) |  |  | 14 (60.9) | 19 (73.1) |  |  |  | 23 (82.1) | 23 (88.5) |  |
| Moderate pain (2,4) |  |  | 8 (34.8) | 4 (15.4) |  |  |  | 5 (17.9) | 1 (3.8) |  |
| Severe pain (6, 8, 10) |  |  | 1 (4.3) | 3 (11.5) |  |  |  | 0 (0.0) | 2 (7.7) |  |
| **Child’s fear (FAS), median (IQR)^3^** | 22 | 26 | 4.7 (1.4–7.6) | 2.7 (0.4–7.5) | 0.198 | 29 | 24 | 3.7 (1.7–5.9) | 4.2 (0.4–4.2) | 0.593 |
| P value for comparison between IG and CG from ^1^ independent samples t-test, ^2^ chi-square test or ^3^ Mann-Whitney U-test.  IG = intervention group, CG = control group, P = p-value, SD = standard deviation, IQR = interquartile range (i.e., 25^th^ - 75^th^ percentiles)  STAI-S = State-Trait Anxiety Inventory, VRSS = The Verbal Rating Scale for Stress Analysis, VAS = The Visual Analogue Scale, WBS = The Wong-Baker FACES^®^ Pain Rating Scale,  FAS = The Facial Affective Scale | | | | | | | | | | |
