# Supplementary material for: Effectiveness of a Mobile App Intervention for Preparing Preschool Children and Parents for Day Surgery: Randomized Controlled Trial
Source: J Med Internet Res. 2023 Sep 29;25:e46989. doi: 10.2196/46989 (PMC10576237; doi:10.2196/46989)
Supplement: Multimedia Appendix 3 [file jmir_v25i1e46989_app3.docx]

**Multimedia Appendix 3**

**Table S3.** Comparison of parent's anxiety, stress and children`s pain and fear between the IG and CG in **the assessments after the day surgery**.

|  | **In the hospital** | | | | | **At home** | | | | |
| --- | --- | --- | --- | --- | --- | --- | --- | --- | --- | --- |
|  | N | | Group | |  |  | |  | |  |
|  | IG | CG | IG | CG | P | IG | CG | IG | CG | P |
| **Parent’s anxiety (STAI-S), mean (SD)^1^** | n.a. | n.a. |  |  |  | 24 | 17 | 28.1 (6.9) | 30.2 (7.1) | 0.344 |
| **Parent’s anxiety categories, n (%)^2^** | n.a. | n.a. |  |  |  |  |  |  |  | 0.629 |
| Mild (20–39) |  |  |  |  |  |  |  | 21 (87.5) | 16 (94.1) |  |
| Moderate (40–59) |  |  |  |  |  |  |  | 3 (12.5) | 1 (5.9) |  |
| Intense (60–80) |  |  |  |  |  |  |  | 0 (0.0) | 0 (0.0) |  |
| **Parent’s stress (VRSS), n (%)^2^** | 32 | 26 |  |  | >0.99 | 24 | 17 |  |  | 0.045 |
| No stress (0) |  |  | 15 (46.9) | 13 (50.0) |  |  |  | 7 (29.2) | 7 (41.2) |  |
| Mild stress (1) |  |  | 13 (40.6) | 10 (38.5) |  |  |  | 17 (70.8) | 7 (41.2) |  |
| Moderate to intense stress (2–5) |  |  | 4 (12.5) | 3 (11.5) |  |  |  | 0 (0.0) | 3 (17.6) |  |
| **Child’s pain, evaluated by parent (VAS), median (IQR)^3^** | 31 | 26 | 1.8 (0.4–2.4) | 1.6 (0.2–3.6) | 0.724 | 20 | 15 | 1.4 (0.5–3.4) | 1.5 (0.5–2.3) | 0.837 |
| **Child’s pain, evaluated by nurse (VAS), median (IQR)^3^** | 32 | 26 | 0.7 (0.0–1.9) | 1.3 (0.0–3.1) | 0.541 | n.a. | n.a. |  |  |  |
| **Child’s pain, evaluated by child (WBS), n (%)^2^** | 28 | 26 |  |  | 0.470 | 20 | 13 |  |  | 0.719 |
| No pain (0) |  |  | 12 (40.0) | 12 (50.0) |  |  |  | 8 (40.0) | 6 (46.2) |  |
| Moderate pain (2,4) |  |  | 14 (46.7) | 7 (29.2) |  |  |  | 9 (45.0) | 4 (30.7) |  |
| Severe pain (6, 8, 10) |  |  | 4 (13.3) | 5 (20.8) |  |  |  | 3 (15.0) | 3 (23.1) |  |
| **Child’s fear (FAS), median (IQR)^3^** | 30 | 25 | 3.7 (0.4–4.7) | 3.7 (1.1–5.9) | 0.616 | 22 | 16 | 0.4 (0.4–4.0) | 1.1 (0.4–5.4) | 0.805 |
| P value for comparison between IG and CG from ^1^ independent samples t-test, ^2^ chi-square test or ^3^ Mann-Whitney U-test.  IG = intervention group, CG = control group, P = p-value, SD = standard deviation, IQR = interquartile range (i.e. 25^th^ - 75^th^ percentiles)  STAI-S = State-Trait Anxiety Inventory, VRSS = The Verbal Rating Scale for Stress analysis, VAS = The Visual Analogue Scale, WBS = The Wong-Baker FACES^®^ Pain Rating scale, FAS = The Facial Affective Scale | | | | | | | | | | |
