# Supplementary material for: Effectiveness of a Mobile App Intervention for Preparing Preschool Children and Parents for Day Surgery: Randomized Controlled Trial
Source: J Med Internet Res. 2023 Sep 29;25:e46989. doi: 10.2196/46989 (PMC10576237; doi:10.2196/46989)
Supplement: Multimedia Appendix 4 [file jmir_v25i1e46989_app4.docx]

**Multimedia Appendix 4**

**Table S4.** Comparison of parent's anxiety, stress and children`s pain and fear from before to after day surgery, **assessments at home.**

|  | **IG** | | | | **CG** | | | |
| --- | --- | --- | --- | --- | --- | --- | --- | --- |
|  | N | Before | After | P | N | Before | After | P |
|  |  |  |  |  |  |  |  |  |
| **Parent’s anxiety (STAI-S), mean (SD)^1^** | 22 | 35.6 (10.1) | 28.7 (6.9) | 0.003 | 17 | 37.2 (10.7) | 30.2 (7.1) | 0.002 |
| **Parent’s anxiety categories, n (%)^2^** |  |  |  | 0.375 |  |  |  | 0.063 |
| Mild (20–39) |  | 16 (72.7) | 19 (86.4) |  |  | 11 (64.7) | 16 (94.1) |  |
| Moderate (40–59) |  | 6 (27.3) | 3 (13.6) |  |  | 6 (35.3) | 1 (5.9) |  |
| Intense (60–80) |  | 0 (0.0) | 0 (0.0) |  |  | 0 (0.0) | 0 (0.0) |  |
| **Parent’s stress (VRSS), n (%)^2^** | 22 |  |  | 0.003 | 17 |  |  | 0.004 |
| No stress (0) |  | 1 (4.5) | 7 (31.8) |  |  | 1 (5.8) | 7 (41.2) |  |
| Mild stress (1) |  | 13 (59.1) | 15 (68.2) |  |  | 8 (47.1) | 7 (41.2) |  |
| Moderate to intense stress (2–5) |  | 8 (36.4) | 0 (0.0) |  |  | 8 (47.1) | 3 (17.6) |  |
| **Child’s pain, evaluated by parent (VAS), median (IQR)^3^** | 16 | 0.2 (0.0–0.8) | 1.3 (0.3–3.7) | 0.055 | 15 | 0.0 (0.0–0.6) | 1.5 (0.5–2.3) | <0.001 |
| **Child’s pain, evaluated by child (WBS), n (%)^2^** | 17 |  |  | 0.227 | 13 |  |  | 0.289 |
| No pain (0) |  | 9 (52.9) | 6 (35.3) |  |  | 8 (61.5) | 6 (46.2) |  |
| Moderate pain (2, 4) |  | 7 (41.2) | 8 (47.1) |  |  | 3 (23.1) | 4 (30.7) |  |
| Severe pain (6, 8, 10) |  | 1 (5.9) | 3 (17.6) |  |  | 2 (15.4) | 3 (23.1) |  |
| **Child’s fear (FAS), median (IQR)^3^** | 17 | 4.7 (1.1–7.7) | 0.4 (0.4–3.7) | 0.006 | 16 | 1.7 (0.4–5.9) | 1.1 (0.4–5.4) | 0.435 |
| P value for comparison between before and after surgery from ^1^ paired samples t-test, ^2^ sign test or ^3^ Wilcoxon signed-rank test.  IG = intervention group, CG = control group, P = p-value, SD = standard deviation, IQR = interquartile range (i.e. 25^th^ - 75^th^ percentiles)  STAI-S = State-Trait Anxiety Inventory, VRSS =The Verbal Rating Scale for Stress analysis, VAS = The Visual Analogue Scale, WBS = The Wong-Baker FACES^®^ Pain Rating scale, FAS = The Facial Affective Scale, | | | | | | | | |
