# Supplementary material for: Effectiveness of a Mobile App Intervention for Preparing Preschool Children and Parents for Day Surgery: Randomized Controlled Trial
Source: J Med Internet Res. 2023 Sep 29;25:e46989. doi: 10.2196/46989 (PMC10576237; doi:10.2196/46989)
Supplement: Multimedia Appendix 5 [file jmir_v25i1e46989_app5.pdf]

# CONSORT-EHEALTH (V 1.6.1) - Submission/Publication Form

The CONSORT-EHEALTH checklist is intended for authors of randomized trials evaluating web-based and Internet-based applications/interventions, including mobile interventions, electronic games (incl multiplayer games), social media, certain telehealth applications, and other interactive and/or networked electronic applications. Some of the items (e.g. all subitems under item 5 - description of the intervention) may also be applicable for other study designs.

The goal of the CONSORT EHEALTH checklist and guideline is to be
a) a guide for reporting for authors of RCTs,
b) to form a basis for appraisal of an ehealth trial (in terms of validity)

CONSORT-EHEALTH items/subitems are MANDATORY reporting items for studies published in the Journal of Medical Internet Research and other journals / scientific societies endorsing the checklist.

Items numbered 1., 2., 3., 4a., 4b etc are original CONSORT or CONSORT-NPT (non-pharmacologic treatment) items.
Items with Roman numerals (i., ii, iii, iv etc.) are CONSORT-EHEALTH extensions/clarifications.

As the CONSORT-EHEALTH checklist is still considered in a formative stage, we would ask that you also RATE ON A SCALE OF 1-5 how important/useful you feel each item is FOR THE PURPOSE OF THE CHECKLIST and reporting guideline (optional).

Mandatory reporting items are marked with a red *.
In the textboxes, either copy & paste the relevant sections from your manuscript into this form - please include any quotes from your manuscript in QUOTATION MARKS,
or answer directly by providing additional information not in the manuscript, or elaborating on why the item was not relevant for this study.

YOUR ANSWERS WILL BE PUBLISHED AS A SUPPLEMENTARY FILE TO YOUR PUBLICATION IN JMIR AND ARE CONSIDERED PART OF YOUR PUBLICATION (IF ACCEPTED).
Please fill in these questions diligently. Information will not be copyedited, so please use proper spelling and grammar, use correct capitalization, and avoid abbreviations.

DO NOT FORGET TO SAVE AS PDF _AND_ CLICK THE SUBMIT BUTTON SO YOUR ANSWERS ARE IN OUR DATABASE !!!

Citation Suggestion (if you append the pdf as Appendix we suggest to cite this paper in the caption):
Eysenbach G, CONSORT-EHEALTH Group
CONSORT-EHEALTH: Improving and Standardizing Evaluation Reports of Web-based and Mobile Health Interventions
J Med Internet Res 2011;13(4):e126

URL: http://www.jmir.org/2011/4/e126/
doi: 10.2196/jmir.1923
PMID: 22209829

heli.kerimaa@gmail.com Vaihda tiliä

Ei jaettu

Luonnos tallennettu

* Pakollinen kysymys

Your name *

First Last

Heli Kerimaa

Primary Affiliation (short), City, Country *

University of Toronto, Toronto, Canada

University of Oulu, Oulu, Finland

Your e-mail address *

abc@gmail.com

heli.kerimaa@oulu.fi

Title of your manuscript *

Provide the (draft) title of your manuscript.

Effectiveness of a mobile application intervention for preparing preschool children and parents for day surgery: a randomized controlled trial

Name of your App/Software/Intervention *

If there is a short and a long/alternate name, write the short name first and add the long name in brackets.

BuddyCare

Evaluated Version (if any)

e.g. "V1", "Release 2017-03-01", "Version 2.0.27913"

Oma vastauksesi

Language(s) *

What language is the intervention/app in? If multiple languages are available, separate by comma (e.g. "English, French")

Finnish

URL of your Intervention Website or App

e.g. a direct link to the mobile app on app in appstore (itunes, Google Play), or URL of the website. If the intervention is a DVD or hardware, you can also link to an Amazon page.

https://www.buddyhealthcare.com/en/

URL of an image/screenshot (optional)

Oma vastauksesi

Accessibility *

Can an enduser access the intervention presently?

- ○ access is free and open
- ◉ access only for special usergroups, not open
- ○ access is open to everyone, but requires payment/subscription/in-app purchases
- ○ app/intervention no longer accessible
- ○ Muu:

Primary Medical Indication/Disease/Condition *

e.g. "Stress", "Diabetes", or define the target group in brackets after the condition, e.g. "Autism (Parents of children with)", "Alzheimers (Informal Caregivers of)"

Preparation for day surgery

Primary Outcomes measured in trial *

comma-separated list of primary outcomes reported in the trial

Parents anxiety

Secondary/other outcomes

Are there any other outcomes the intervention is expected to affect?

Parents stress and children pain and fear

Recommended "Dose" *

What do the instructions for users say on how often the app should be used?

- (x) Approximately Daily
- ( ) Approximately Weekly
- ( ) Approximately Monthly
- ( ) Approximately Yearly
- ( ) "as needed"
- ( ) Muu:

Approx. Percentage of Users (starters) still using the app as recommended after 3 months *

- (x) unknown / not evaluated
- ( ) 0-10%
- ( ) 11-20%
- ( ) 21-30%
- ( ) 31-40%
- ( ) 41-50%
- ( ) 51-60%
- ( ) 61-70%
- ( ) 71%-80%
- ( ) 81-90%
- ( ) 91-100%
- ( ) Muu:

Overall, was the app/intervention effective? *

- ( ) yes: all primary outcomes were significantly better in intervention group vs control
- (•) partly: SOME primary outcomes were significantly better in intervention group vs control
- ( ) no statistically significant difference between control and intervention
- ( ) potentially harmful: control was significantly better than intervention in one or more outcomes
- ( ) inconclusive: more research is needed
- ( ) Muu:

Article Preparation Status/Stage *

At which stage in your article preparation are you currently (at the time you fill in this form)

- ( ) not submitted yet - in early draft status
- ( ) not submitted yet - in late draft status, just before submission
- ( ) submitted to a journal but not reviewed yet
- (•) submitted to a journal and after receiving initial reviewer comments
- ( ) submitted to a journal and accepted, but not published yet
- ( ) published
- ( ) Muu:

Journal *

If you already know where you will submit this paper (or if it is already submitted), please provide the journal name (if it is not JMIR, provide the journal name under "other")

- ○ not submitted yet / unclear where I will submit this
- ◉ Journal of Medical Internet Research (JMIR)
- ○ JMIR mHealth and UHealth
- ○ JMIR Serious Games
- ○ JMIR Mental Health
- ○ JMIR Public Health
- ○ JMIR Formative Research
- ○ Other JMIR sister journal
- ○ Muu:

Is this a full powered effectiveness trial or a pilot/feasibility trial? *

- ○ Pilot/feasibility
- ◉ Fully powered

Manuscript tracking number *

If this is a JMIR submission, please provide the manuscript tracking number under "other" (The ms tracking number can be found in the submission acknowledgement email, or when you login as author in JMIR. If the paper is already published in JMIR, then the ms tracking number is the four-digit number at the end of the DOI, to be found at the bottom of each published article in JMIR)

- ◉ no ms number (yet) / not (yet) submitted to / published in JMIR
- ○ Muu:

TITLE AND ABSTRACT

1a) TITLE: Identification as a randomized trial in the title

1a) Does your paper address CONSORT item 1a? *

I.e does the title contain the phrase "Randomized Controlled Trial"? (if not, explain the reason under "other")

- (x) yes
- ( ) Muu:

1a-i) Identify the mode of delivery in the title

Identify the mode of delivery. Preferably use “web-based” and/or “mobile” and/or “electronic game” in the title. Avoid ambiguous terms like “online”, “virtual”, “interactive”. Use “Internet-based” only if Intervention includes non-web-based Internet components (e.g. email), use “computer-based” or “electronic” only if offline products are used. Use “virtual” only in the context of “virtual reality” (3-D worlds). Use “online” only in the context of “online support groups”. Complement or substitute product names with broader terms for the class of products (such as “mobile” or “smart phone” instead of “iphone”), especially if the application runs on different platforms.

subitem not at all important

1 ( )

2 ( )

3 ( )

4 ( )

5 (x)

essential

Tyhjennä valinta

Does your paper address subitem 1a-i? *

Copy and paste relevant sections from manuscript title (include quotes in quotation marks "like this" to indicate direct quotes from your manuscript), or elaborate on this item by providing additional information not in the ms, or briefly explain why the item is not applicable/relevant for your study

Effectiveness of a mobile application intervention for preparing preschool children and parents for day surgery: a randomized controlled trial

1a-ii) Non-web-based components or important co-interventions in title

Mention non-web-based components or important co-interventions in title, if any (e.g., “with telephone support”).

subitem not at all important

1 ○

2 ○

3 ○

4 ○

5 ◉

essential

Tyhjennä valinta

Does your paper address subitem 1a-ii?

Copy and paste relevant sections from manuscript title (include quotes in quotation marks "like this" to indicate direct quotes from your manuscript), or elaborate on this item by providing additional information not in the ms, or briefly explain why the item is not applicable/relevant for your study

Effectiveness of a mobile application intervention for preparing preschool children and parents for day surgery: a randomized controlled trial

1a-iii) Primary condition or target group in the title

Mention primary condition or target group in the title, if any (e.g., “for children with Type I Diabetes”) Example: A Web-based and Mobile Intervention with Telephone Support for Children with Type I Diabetes: Randomized Controlled Trial

subitem not at all important

- 1 ○
- 2 ○
- 3 ○
- 4 ○
- 5 ◉

essential

Tyhjennä valinta

Does your paper address subitem 1a-iii? *

Copy and paste relevant sections from manuscript title (include quotes in quotation marks "like this" to indicate direct quotes from your manuscript), or elaborate on this item by providing additional information not in the ms, or briefly explain why the item is not applicable/relevant for your study

Effectiveness of a mobile application intervention for preparing preschool children and parents for day surgery: a randomized controlled trial

1b) ABSTRACT: Structured summary of trial design, methods, results, and conclusions

NPT extension: Description of experimental treatment, comparator, care providers, centers, and blinding status.

1b-i) Key features/functionalities/components of the intervention and comparator in the METHODS section of the ABSTRACT

Mention key features/functionalities/components of the intervention and comparator in the abstract. If possible, also mention theories and principles used for designing the site. Keep in mind the needs of systematic reviewers and indexers by including important synonyms. (Note: Only report in the abstract what the main paper is reporting. If this information is missing from the main body of text, consider adding it)

subitem not at all important

1 ○

2 ○

3 ○

4 ○

5 ◉

essential

Tyhjennä valinta

Does your paper address subitem 1b-i? *

Copy and paste relevant sections from the manuscript abstract (include quotes in quotation marks "like this" to indicate direct quotes from your manuscript), or elaborate on this item by providing additional information not in the ms, or briefly explain why the item is not applicable/relevant for your study

"Parents of children (ages 2-6 years) who were in a queue for elective day surgery were randomized into intervention (IG, n=36) and control (CG, n=34) groups. The CG received routine preparation while the IG was prepared using a mobile application."

1b-ii) Level of human involvement in the METHODS section of the ABSTRACT

Clarify the level of human involvement in the abstract, e.g., use phrases like “fully automated” vs. “therapist/nurse/care provider/physician-assisted” (mention number and expertise of providers involved, if any). (Note: Only report in the abstract what the main paper is reporting. If this information is missing from the main body of text, consider adding it)

subitem not at all important

1 ○

2 ○

3 ○

4 ○

5 ◉

essential

Tyhjennä valinta

Does your paper address subitem 1b-ii?

Copy and paste relevant sections from the manuscript abstract (include quotes in quotation marks "like this" to indicate direct quotes from your manuscript), or elaborate on this item by providing additional information not in the ms, or briefly explain why the item is not applicable/relevant for your study

"Parents of children (ages 2-6 years) who were in a queue for elective day surgery were randomized into intervention (IG, n=36) and control (CG, n=34) groups. The CG received routine preparation while the IG was prepared using a mobile application."

1b-iii) Open vs. closed, web-based (self-assessment) vs. face-to-face assessments in the METHODS section of the ABSTRACT

Mention how participants were recruited (online vs. offline), e.g., from an open access website or from a clinic or a closed online user group (closed usergroup trial), and clarify if this was a purely web-based trial, or there were face-to-face components (as part of the intervention or for assessment). Clearly say if outcomes were self-assessed through questionnaires (as common in web-based trials). Note: In traditional offline trials, an open trial (open-label trial) is a type of clinical trial in which both the researchers and participants know which treatment is being administered. To avoid confusion, use “blinded” or “unblinded” to indicated the level of blinding instead of “open”, as “open” in web-based trials usually refers to “open access” (i.e. participants can self-enrol). (Note: Only report in the abstract what the main paper is reporting. If this information is missing from the main body of text, consider adding it)

subitem not at all important

1 ○

2 ○

3 ○

4 ○

5 ◉

essential

Tyhjennä valinta

Does your paper address subitem 1b-iii?

Copy and paste relevant sections from the manuscript abstract (include quotes in quotation marks "like this" to indicate direct quotes from your manuscript), or elaborate on this item by providing additional information not in the ms, or briefly explain why the item is not applicable/relevant for your study

"Parents and children's outcomes were measured using validated scales at four different points: at home (T1, T4) and hospital (T2, T3), before and after surgery. Group differences were analyzed using statistical methods suitable for the material."

1b-iv) RESULTS section in abstract must contain use data

Report number of participants enrolled/assessed in each group, the use/uptake of the intervention (e.g., attrition/adherence metrics, use over time, number of logins etc.), in addition to primary/secondary outcomes. (Note: Only report in the abstract what the main paper is reporting. If this information is missing from the main body of text, consider adding it)

subitem not at all important

1 ○

2 ○

3 ○

4 ○

5 ◉

essential

Tyhjennä valinta

Does your paper address subitem 1b-iv?

Copy and paste relevant sections from the manuscript abstract (include quotes in quotation marks "like this"  to indicate direct quotes from your manuscript), or elaborate on this item by providing additional information not in the ms, or briefly explain why the item is not applicable/relevant for your study

"Before surgery, parents in both groups experienced mild anxiety and stress, which decreased after the surgery. Parental anxiety did not differ between the groups preoperatively (P=.78) or postoperatively (P=.63). Both groups had less anxiety at home after surgery than before, with the IG showing a significant decrease (P=.003) and the CG also improving (P=.002). Preoperatively at home, most parents in both groups experienced no stress or mild stress (P=.61). Preoperatively in the hospital, the parents in both groups experienced mild stress; however, parents in the IG experienced more stress during this phase (P=.02). Postoperatively in the hospital, most parents in both groups experienced no stress (P>.99). Parents in the IG experienced less stress postoperative compared to the CG, with a significant difference between the groups (P=.05). Both groups showed decreased stress levels from before to after surgery (IG P=.003 and CG P=.004) within each group. The child's pain before surgery was rated mild in both groups by parents and children and more intense after the surgery. According to a median of VAS (The Visual Analogue Scale), there were no statistically significant differences in p-values in children's pain between groups and measurement points preoperatively at home (P=.25), preoperatively in the hospital (P=.98) and postoperatively in the hospital (P=.72). The child's fear decreased more in the IG (0.4) than in the CG (1.1) after surgery at home. The median of FAS (The Facial Affective Scale) did not differ between the IG and CG preoperatively (P=.20), in the hospital (P=.59) or postoperatively in the hospital (P=.62) at home (P=.81). "

1b-v) CONCLUSIONS/DISCUSSION in abstract for negative trials

Conclusions/Discussions in abstract for negative trials: Discuss the primary outcome - if the trial is negative (primary outcome not changed), and the intervention was not used, discuss whether negative results are attributable to lack of uptake and discuss reasons. (Note: Only report in the abstract what the main paper is reporting. If this information is missing from the main body of text, consider adding it)

subitem not at all important

1 ○

2 ○

3 ○

4 ○

5 ◉

essential

Tyhjennä valinta

Does your paper address subitem 1b-v?

Copy and paste relevant sections from the manuscript abstract (include quotes in quotation marks "like this" to indicate direct quotes from your manuscript), or elaborate on this item by providing additional information not in the ms, or briefly explain why the item is not applicable/relevant for your study

"Although to the study, the mobile application intervention did not reduce anxiety or pain levels. However, it was observed that parents of the intervention group experienced statistically significant heightened stress levels before surgery at the hospital, which decreased statistically significantly after the surgery at home. Additionally, fear levels in children of the intervention group decreased over time, while no significant change was observed in the control group."

INTRODUCTION

2a) In INTRODUCTION: Scientific background and explanation of rationale

2a-i) Problem and the type of system/solution

Describe the problem and the type of system/solution that is object of the study: intended as stand-alone intervention vs. incorporated in broader health care program? Intended for a particular patient population? Goals of the intervention, e.g., being more cost-effective to other interventions, replace or complement other solutions? (Note: Details about the intervention are provided in “Methods” under 5)

subitem not at all important

1 ○

2 ○

3 ○

4 ○

5 ◉

essential

Tyhjennä valinta

Does your paper address subitem 2a-i? *

Copy and paste relevant sections from the manuscript (include quotes in quotation marks "like this" to indicate direct quotes from your manuscript), or elaborate on this item by providing additional information not in the ms, or briefly explain why the item is not applicable/relevant for your study

"Day surgery affords families the possibility of a quick return home and to everyday life, usually within one day [1]. Globally, the number of day surgery procedures for children has increased significantly. In the United States, more than five million pediatric patients are estimated to participate in planned day surgery or diagnostic procedures every year [2,3]. Other countries have also witnessed a growing number of pediatric patients over recent years [4]. As pediatric day surgery rates grow, parents need to be educated about their role in day surgery and what happens at different stages of the surgical process [4]. Their situation is rather complicated because parents have several roles in preparing their child for day surgery: they bear responsibility, make important decisions, and support the child through the process [5,6]. It is therefore understandable that parents often experience anxiety and stress [3,7,8], which are natural responses to a new, challenging situation [9]. Preschool children (2 to 6 years old) also find day surgery scary [10,11]. A child's fear is often related to the unknown [11] and the worry of being separated from their parents [12,13]. The rapid development of information and communication technologies (ICT) and the increased prevalence of smartphones create new opportunities for using web-based or digital programs to prepare patients for day surgery [14,15]. The effectiveness of mobile application interventions for preschool children and parents has already been studied in chronically ill children [16], adults [17,18], and health monitoring [19]. In the older age group (9-17 years old), studies have shown that playing computer games before surgery can be effective in reducing children's separation and pre-surgery anxiety levels

2a-ii) Scientific background, rationale: What is known about the (type of) system

Scientific background, rationale: What is known about the (type of) system that is the object of the study (be sure to discuss the use of similar systems for other conditions/diagnoses, if appropiate), motivation for the study, i.e. what are the reasons for and what is the context for this specific study, from which stakeholder viewpoint is the study performed, potential impact of findings [2]. Briefly justify the choice of the comparator.

subitem not at all important

1 ○

2 ○

3 ○

4 ○

5 ◉

essential

Tyhjennä valinta

Does your paper address subitem 2a-ii? *

Copy and paste relevant sections from the manuscript (include quotes in quotation marks "like this" to indicate direct quotes from your manuscript), or elaborate on this item by providing additional information not in the ms, or briefly explain why the item is not applicable/relevant for your study

"Preparation for pediatric day surgery sets out to improve parents' understanding of the surgery to be performed and improve cooperation between the child and health care personnel. According to earlier studies, some 50–70 % of parents experience anxiety [21] and stress [22] about their child's day surgery [23,24]. This anxiety may be transferred to the child, amplifying their feelings of pain and fear [3,7,8]. Parental anxiety and stress are caused by uncertainty, lack of control in a new situation [9], and various fears about pediatric surgery [5,6]. Parents' lack of knowledge can lead them to experience guilt, ignorance, separation anxiety, and feeling out of control [9]. Research has also shown that 65%-80% of children experience anxiety or fear [25] prior to surgery [10,26,27]. Up to 30% of children also suffer moderate or significant pain [28] as a result of day surgery

2b) In INTRODUCTION: Specific objectives or hypotheses

Does your paper address CONSORT subitem 2b? *

Copy and paste relevant sections from the manuscript (include quotes in quotation marks "like this" to indicate direct quotes from your manuscript), or elaborate on this item by providing additional information not in the ms, or briefly explain why the item is not applicable/relevant for your study

"The aim of this study was to evaluate the effectiveness of a mobile application intervention for preparing preschool children and their parents for pediatric day surgery. In this study, children's pain and fear, and parents` anxiety and stress, were measured at home and hospital before and after day surgery. The first hypothesis was that parents of the intervention group would show lower levels of anxiety and stress at home and hospital before and after the day surgery than parents in the control group. The second hypothesis was that preschool children of the intervention group would show lower levels of pain and fear at home and hospital before and after the day surgery than preschool children in the control group."

METHODS

3a) Description of trial design (such as parallel, factorial) including allocation ratio

Does your paper address CONSORT subitem 3a? *

Copy and paste relevant sections from the manuscript (include quotes in quotation marks "like this" to indicate direct quotes from your manuscript), or elaborate on this item by providing additional information not in the ms, or briefly explain why the item is not applicable/relevant for your study

"This study employed a two-armed randomized controlled trial (RCT) design."

3b) Important changes to methods after trial commencement (such as eligibility criteria), with reasons

Does your paper address CONSORT subitem 3b? *

Copy and paste relevant sections from the manuscript (include quotes in quotation marks "like this" to indicate direct quotes from your manuscript), or elaborate on this item by providing additional information not in the ms, or briefly explain why the item is not applicable/relevant for your study

"There were no changes made to the methods after the trial commenced."

3b-i) Bug fixes, Downtimes, Content Changes

Bug fixes, Downtimes, Content Changes: ehealth systems are often dynamic systems. A description of changes to methods therefore also includes important changes made on the intervention or comparator during the trial (e.g., major bug fixes or changes in the functionality or content) (5-iii) and other "unexpected events" that may have influenced study design such as staff changes, system failures/downtimes, etc. [2].

subitem not at all important

1 ○

2 ○

3 ○

4 ○

5 ◉

essential

Tyhjennä valinta

Does your paper address subitem 3b-i?

Copy and paste relevant sections from the manuscript (include quotes in quotation marks "like this" to indicate direct quotes from your manuscript), or elaborate on this item by providing additional information not in the ms, or briefly explain why the item is not applicable/relevant for your study

"There were no changes made to the methods after the trial commenced."

4a) Eligibility criteria for participants

Does your paper address CONSORT subitem 4a? *

Copy and paste relevant sections from the manuscript (include quotes in quotation marks "like this" to indicate direct quotes from your manuscript), or elaborate on this item by providing additional information not in the ms, or briefly explain why the item is not applicable/relevant for your study

"The study was conducted between January 2018 and May 2020 at the Pediatric Day Surgical Department of one University Hospital in Finland. Participants included the parents of preschool children (2–6 years old) who were due to undergo elective day surgery under general anesthesia. The other inclusion criteria are listed in Table 1. The mobile application was available for use for 3-4 weeks before the operation, so that parents had sufficient time to use the mobile application to prepare for their child's surgery."

4a-i) Computer / Internet literacy

Computer / Internet literacy is often an implicit "de facto" eligibility criterion - this should be explicitly clarified.

subitem not at all important

1 ○

2 ○

3 ○

4 ○

5 ●

essential

Tyhjennä valinta

Does your paper address subitem 4a-i?

Copy and paste relevant sections from the manuscript (include quotes in quotation marks "like this" to indicate direct quotes from your manuscript), or elaborate on this item by providing additional information not in the ms, or briefly explain why the item is not applicable/relevant for your study

"Parents with access to an Android or iOS phone, iPad, or an internet browser."

4a-ii) Open vs. closed, web-based vs. face-to-face assessments:

Open vs. closed, web-based vs. face-to-face assessments: Mention how participants were recruited (online vs. offline), e.g., from an open access website or from a clinic, and clarify if this was a purely web-based trial, or there were face-to-face components (as part of the intervention or for assessment), i.e., to what degree got the study team to know the participant. In online-only trials, clarify if participants were quasi-anonymous and whether having multiple identities was possible or whether technical or logistical measures (e.g., cookies, email confirmation, phone calls) were used to detect/prevent these.

subitem not at all important

1 ○

2 ○

3 ○

4 ○

5 ◉

essential

Tyhjennä valinta

Does your paper address subitem 4a-ii? *

Copy and paste relevant sections from the manuscript (include quotes in quotation marks "like this" to indicate direct quotes from your manuscript), or elaborate on this item by providing additional information not in the ms, or briefly explain why the item is not applicable/relevant for your study

"The application contained information for both parents and children, presented in various formats including video, photo and written instructions. Contents included images of the day surgery unit, instructions for the surgery and pain care, information on how to get to the hospital and the ward, notifications and the necessary forms. After registering on the application, users' information was passed on to the hospital, allowing health care professionals (HCPs) to keep track of a family's preparation for the surgery. The application also included a video describing the progression through surgery at the hospital (arrival to discharge, along with information about some non-pharmacological pain management methods), from both the child's and parent's perspective. This version did not have a chat feature."

4a-iii) Information giving during recruitment

Information given during recruitment. Specify how participants were briefed for recruitment and in the informed consent procedures (e.g., publish the informed consent documentation as appendix, see also item X26), as this information may have an effect on user self-selection, user expectation and may also bias results.

subitem not at all important

1 ○

2 ○

3 ○

4 ○

5 ◉

essential

Tyhjennä valinta

Does your paper address subitem 4a-iii?

Copy and paste relevant sections from the manuscript (include quotes in quotation marks "like this" to indicate direct quotes from your manuscript), or elaborate on this item by providing additional information not in the ms, or briefly explain why the item is not applicable/relevant for your study

"A pediatric surgeon assessed the need for surgery and put the child in the surgery queue. After planning the surgery, the schedule went to secretaries who had been trained by the researcher to facilitate the study. Patients suitable for the study were selected from the schedule according to the selection criteria. Each month, the secretaries provided the researcher with information about the number of study families identified and their details. The researcher then called the families and asked about their willingness to participate in the study. After receiving oral consent, the secretaries sent the families written informed consent forms relating to the study, instructions, and questionnaires. The parents were informed about the study both verbally and in writing. Families in the IG also received instructions for logging in to the mobile application and using the application, through which they could access instructions for preparing for the surgery. The CG received the conventional preparation instructions."

4b) Settings and locations where the data were collected

Does your paper address CONSORT subitem 4b? *

Copy and paste relevant sections from the manuscript (include quotes in quotation marks "like this" to indicate direct quotes from your manuscript), or elaborate on this item by providing additional information not in the ms, or briefly explain why the item is not applicable/relevant for your study

"The study was conducted between January 2018 and May 2020 at the Pediatric Day Surgical Department of one University Hospital in Finland. Participants included the parents of preschool children (2–6 years old) who were due to undergo elective day surgery under general anesthesia. The other inclusion criteria are listed in Table 1. The mobile application was available for use for 3-4 weeks before the operation, so that parents had sufficient time to use the mobile application to prepare for their child's surgery."

4b-i) Report if outcomes were (self-)assessed through online questionnaires

Clearly report if outcomes were (self-)assessed through online questionnaires (as common in web-based trials) or otherwise.

subitem not at all important

1 ○

2 ○

3 ○

4 ○

5 ◉

essential

Tyhjennä valinta

Does your paper address subitem 4b-i? *

Copy and paste relevant sections from the manuscript (include quotes in quotation marks "like this" to indicate direct quotes from your manuscript), or elaborate on this item by providing additional information not in the ms, or briefly explain why the item is not applicable/relevant for your study

"The forms sent home to parents were coded using the same code that was used for T2 and T3. The questionnaires designed for parents with children were organized in a manner such that each page featured only one meter and provided concise instructions for completing it."

4b-ii) Report how institutional affiliations are displayed

Report how institutional affiliations are displayed to potential participants [on ehealth media], as affiliations with prestigious hospitals or universities may affect volunteer rates, use, and reactions with regards to an intervention.(Not a required item – describe only if this may bias results)

subitem not at all important

1 (selected)

2

3

4

5

essential

Tyhjennä valinta

Does your paper address subitem 4b-ii?

Copy and paste relevant sections from the manuscript (include quotes in quotation marks "like this" to indicate direct quotes from your manuscript), or elaborate on this item by providing additional information not in the ms, or briefly explain why the item is not applicable/relevant for your study

Oma vastauksesi

5) The interventions for each group with sufficient details to allow replication, including how and when they were actually administered

5-i) Mention names, credential, affiliations of the developers, sponsors, and owners

Mention names, credential, affiliations of the developers, sponsors, and owners [6] (if authors/evaluators are owners or developer of the software, this needs to be declared in a “Conflict of interest” section or mentioned elsewhere in the manuscript).

subitem not at all important

1 ○

2 ○

3 ○

4 ○

5 ◉

essential

Tyhjennä valinta

Does your paper address subitem 5-i?

Copy and paste relevant sections from the manuscript (include quotes in quotation marks "like this" to indicate direct quotes from your manuscript), or elaborate on this item by providing additional information not in the ms, or briefly explain why the item is not applicable/relevant for your study

Section Application use by parents

5-ii) Describe the history/development process

Describe the history/development process of the application and previous formative evaluations (e.g., focus groups, usability testing), as these will have an impact on adoption/use rates and help with interpreting results.

subitem not at all important

1 ○

2 ○

3 ○

4 ○

5 ◉

essential

Tyhjennä valinta

Does your paper address subitem 5-ii?

Copy and paste relevant sections from the manuscript (include quotes in quotation marks "like this" to indicate direct quotes from your manuscript), or elaborate on this item by providing additional information not in the ms, or briefly explain why the item is not applicable/relevant for your study

Section Developing and piloting the mobile application

### 5-iii) Revisions and updating

Revisions and updating. Clearly mention the date and/or version number of the application/intervention (and comparator, if applicable) evaluated, or describe whether the intervention underwent major changes during the evaluation process, or whether the development and/or content was “frozen” during the trial. Describe dynamic components such as news feeds or changing content which may have an impact on the replicability of the intervention (for unexpected events see item 3b).

subitem not at all important

- 1 (selected)
- 2
- 3
- 4
- 5

essential

Tyhjennä valinta

### Does your paper address subitem 5-iii?

Copy and paste relevant sections from the manuscript (include quotes in quotation marks "like this" to indicate direct quotes from your manuscript), or elaborate on this item by providing additional information not in the ms, or briefly explain why the item is not applicable/relevant for your study

Oma vastauksesi

5-iv) Quality assurance methods

Provide information on quality assurance methods to ensure accuracy and quality of information provided [1], if applicable.

subitem not at all important

1 ○

2 ○

3 ○

4 ○

5 ◉

essential

Tyhjennä valinta

Does your paper address subitem 5-iv?

Copy and paste relevant sections from the manuscript (include quotes in quotation marks "like this" to indicate direct quotes from your manuscript), or elaborate on this item by providing additional information not in the ms, or briefly explain why the item is not applicable/relevant for your study

Section Validity, reliability, and rigor

5-v) Ensure replicability by publishing the source code, and/or providing screenshots/screen-capture video, and/or providing flowcharts of the algorithms used

Ensure replicability by publishing the source code, and/or providing screenshots/screen-capture video, and/or providing flowcharts of the algorithms used. Replicability (i.e., other researchers should in principle be able to replicate the study) is a hallmark of scientific reporting.

subitem not at all important

1 (selected)

2

3

4

5

essential

Tyhjennä valinta

Does your paper address subitem 5-v?

Copy and paste relevant sections from the manuscript (include quotes in quotation marks "like this" to indicate direct quotes from your manuscript), or elaborate on this item by providing additional information not in the ms, or briefly explain why the item is not applicable/relevant for your study

Oma vastauksesi

5-vi) Digital preservation

Digital preservation: Provide the URL of the application, but as the intervention is likely to change or disappear over the course of the years; also make sure the intervention is archived (Internet Archive, webcitation.org, and/or publishing the source code or screenshots/videos alongside the article). As pages behind login screens cannot be archived, consider creating demo pages which are accessible without login.

subitem not at all important

1 (selected)

2

3

4

5

essential

Tyhjennä valinta

Does your paper address subitem 5-vi?

Copy and paste relevant sections from the manuscript (include quotes in quotation marks "like this" to indicate direct quotes from your manuscript), or elaborate on this item by providing additional information not in the ms, or briefly explain why the item is not applicable/relevant for your study

Oma vastauksesi

5-vii) Access

Access: Describe how participants accessed the application, in what setting/context, if they had to pay (or were paid) or not, whether they had to be a member of specific group. If known, describe how participants obtained “access to the platform and Internet” [1]. To ensure access for editors/reviewers/readers, consider to provide a “backdoor” login account or demo mode for reviewers/readers to explore the application (also important for archiving purposes, see vi).

subitem not at all important

1 ○

2 ○

3 ○

4 ○

5 ◉

essential

Tyhjennä valinta

Does your paper address subitem 5-vii? *

Copy and paste relevant sections from the manuscript (include quotes in quotation marks "like this" to indicate direct quotes from your manuscript), or elaborate on this item by providing additional information not in the ms, or briefly explain why the item is not applicable/relevant for your study

Section Application use by parents

5-viii) Mode of delivery, features/functionalities/components of the intervention and comparator, and the theoretical framework

Describe mode of delivery, features/functionalities/components of the intervention and comparator, and the theoretical framework [6] used to design them (instructional strategy [1], behaviour change techniques, persuasive features, etc., see e.g., [7, 8] for terminology). This includes an in-depth description of the content (including where it is coming from and who developed it) [1],” whether [and how] it is tailored to individual circumstances and allows users to track their progress and receive feedback” [6]. This also includes a description of communication delivery channels and – if computer-mediated communication is a component – whether communication was synchronous or asynchronous [6]. It also includes information on presentation strategies [1], including page design principles, average amount of text on pages, presence of hyperlinks to other resources, etc. [1].

subitem not at all important

1 ○

2 ○

3 ○

4 ○

5 ◉

essential

Tyhjennä valinta

Does your paper address subitem 5-viii? *

Copy and paste relevant sections from the manuscript (include quotes in quotation marks "like this" to indicate direct quotes from your manuscript), or elaborate on this item by providing additional information not in the ms, or briefly explain why the item is not applicable/relevant for your study

Section Developing and piloting the mobile application, Application use by parents

5-ix) Describe use parameters

Describe use parameters (e.g., intended “doses” and optimal timing for use). Clarify what instructions or recommendations were given to the user, e.g., regarding timing, frequency, heaviness of use, if any, or was the intervention used ad libitum.

subitem not at all important

1 (selected)

2

3

4

5

essential

Tyhjennä valinta

Does your paper address subitem 5-ix?

Copy and paste relevant sections from the manuscript (include quotes in quotation marks "like this" to indicate direct quotes from your manuscript), or elaborate on this item by providing additional information not in the ms, or briefly explain why the item is not applicable/relevant for your study

We Assessing user engagement on our application presents a significant challenge. Metrics such as login frequency and session duration are not entirely reliable indicators of user activity. Though we have yet to establish a dependable way of tracking questionnaire completion rates, we have observed that parents have consistently exhibited timely task and survey completion behaviors. This is evidenced by the nearly 100% acknowledgement rates for timeline events.

### 5-x) Clarify the level of human involvement

Clarify the level of human involvement (care providers or health professionals, also technical assistance) in the e-intervention or as co-intervention (detail number and expertise of professionals involved, if any, as well as “type of assistance offered, the timing and frequency of the support, how it is initiated, and the medium by which the assistance is delivered”. It may be necessary to distinguish between the level of human involvement required for the trial, and the level of human involvement required for a routine application outside of a RCT setting (discuss under item 21 – generalizability).

subitem not at all important

1 ○

2 ○

3 ○

4 ○

5 ◉

essential

Tyhjennä valinta

### Does your paper address subitem 5-x?

Copy and paste relevant sections from the manuscript (include quotes in quotation marks "like this" to indicate direct quotes from your manuscript), or elaborate on this item by providing additional information not in the ms, or briefly explain why the item is not applicable/relevant for your study

"The intervention was developed through multi-professional collaboration, taking into account expert input from nurses and physicians involved in pediatric day surgery. The development process also took into consideration that each hospital, family and child is unique. The information provided in the application was easy to understand, using a simple timeline and incorporating the routine preparation material received by the CG. The purpose of the timeline was to inform users about the phases of surgery and give information to families at the relevant time. (Figure 2). "

5-xi) Report any prompts/reminders used

Report any prompts/reminders used: Clarify if there were prompts (letters, emails, phone calls, SMS) to use the application, what triggered them, frequency etc. It may be necessary to distinguish between the level of prompts/reminders required for the trial, and the level of prompts/reminders for a routine application outside of a RCT setting (discuss under item 21 – generalizability).

subitem not at all important

1 ○

2 ○

3 ○

4 ○

5 ◉

essential

Tyhjennä valinta

Does your paper address subitem 5-xi? *

Copy and paste relevant sections from the manuscript (include quotes in quotation marks "like this" to indicate direct quotes from your manuscript), or elaborate on this item by providing additional information not in the ms, or briefly explain why the item is not applicable/relevant for your study

"The application contained information for both parents and children, presented in various formats including video, photo and written instructions. Contents included images of the day surgery unit, instructions for the surgery and pain care, information on how to get to the hospital and the ward, notifications and the necessary forms. After registering on the application, users' information was passed on to the hospital, allowing health care professionals (HCPs) to keep track of a family's preparation for the surgery. The application also included a video describing the progression through surgery at the hospital (arrival to discharge, along with information about some non-pharmacological pain management methods), from both the child's and parent's perspective. This version did not have a chat feature."

5-xii) Describe any co-interventions (incl. training/support)

Describe any co-interventions (incl. training/support): Clearly state any interventions that are provided in addition to the targeted eHealth intervention, as ehealth intervention may not be designed as stand-alone intervention. This includes training sessions and support [1]. It may be necessary to distinguish between the level of training required for the trial, and the level of training for a routine application outside of a RCT setting (discuss under item 21 – generalizability.

subitem not at all important

1 ○

2 ○

3 ○

4 ○

5 ◉

essential

Tyhjennä valinta

Does your paper address subitem 5-xii? *

Copy and paste relevant sections from the manuscript (include quotes in quotation marks "like this" to indicate direct quotes from your manuscript), or elaborate on this item by providing additional information not in the ms, or briefly explain why the item is not applicable/relevant for your study

"The application contained information for both parents and children, presented in various formats including video, photo and written instructions. Contents included images of the day surgery unit, instructions for the surgery and pain care, information on how to get to the hospital and the ward, notifications and the necessary forms. After registering on the application, users' information was passed on to the hospital, allowing health care professionals (HCPs) to keep track of a family's preparation for the surgery. The application also included a video describing the progression through surgery at the hospital (arrival to discharge, along with information about some non-pharmacological pain management methods), from both the child's and parent's perspective. This version did not have a chat feature."

6a) Completely defined pre-specified primary and secondary outcome measures, including how and when they were assessed

Does your paper address CONSORT subitem 6a? *

Copy and paste relevant sections from the manuscript (include quotes in quotation marks "like this" to indicate direct quotes from your manuscript), or elaborate on this item by providing additional information not in the ms, or briefly explain why the item is not applicable/relevant for your study

"On arrival at the hospital, parents brought the written consent form along with the first stage assessment (T1), which they had completed at home. This first baseline assessment included general demographic data, along with parents' self-reported stress and anxiety, and children`s reported pain and fear. After the physician had met the child and made the final decision for surgery, a nurse collected follow-up data in the hospital before the child's procedure (T2) and after the surgery, and before the child was discharged from the hospital (T3). In the second (T2) and third (T3) measurements, parents were asked to rate the stress they experienced. After the child and parents returned home, one parent took the study-related measures for three days at home, 1-3 times a day depending on the assessment (T4). In the second assessment parents self-reported their anxiety and stress and their child's self-reported pain and fear regarding the care (Figure 1). The forms sent home to parents were coded using the same code that was used for T2 and T3. The questionnaires designed for parents with children were organized in a manner such that each page featured only one meter and provided concise

6a-i) Online questionnaires: describe if they were validated for online use and apply CHERRIES items to describe how the questionnaires were designed/deployed

If outcomes were obtained through online questionnaires, describe if they were validated for online use and apply CHERRIES items to describe how the questionnaires were designed/deployed [9].

subitem not at all important

1 ○

2 ○

3 ○

4 ○

5 ◉

essential

Tyhjennä valinta

Does your paper address subitem 6a-i?

Copy and paste relevant sections from manuscript text

"After receiving oral consent, the secretaries sent the families written informed consent forms relating to the study, instructions, and questionnaires. The parents were informed about the study both verbally and in writing. Families in the IG also received instructions for logging in to the mobile application and using the application, through which they could access instructions for preparing for the surgery. The CG received the conventional preparation instructions. On arrival at the hospital, parents brought the written consent form along with the first stage assessment (T1), which they had completed at home. This first baseline assessment included general demographic data, along with parents' self-reported stress and anxiety, and children`s reported pain and fear. After the physician had met the child and made the final decision for surgery, a nurse collected follow-up data in the hospital before the child's procedure (T2) and after the surgery, and before the child was discharged from the hospital (T3). In the second (T2) and third (T3) measurements, parents were asked to rate the stress they experienced. After the child and parents returned home, one parent took the study-related measures for three days at home, 1-3 times a day depending on the assessment (T4). In the second assessment parents self-reported their anxiety and stress and their child's self-reported pain and fear regarding the care (Figure 1). The forms sent home to parents were coded using the same code that was used for T2 and T3. The questionnaires designed for parents with children were organized in a manner such that each page featured only one meter and provided concise instructions for completing it."

6a-ii) Describe whether and how “use” (including intensity of use/dosage) was defined/measured/monitored

Describe whether and how “use” (including intensity of use/dosage) was defined/measured/monitored (logins, logfile analysis, etc.). Use/adoption metrics are important process outcomes that should be reported in any ehealth trial.

subitem not at all important

1 ○

2 ○

3 ○

4 ○

5 ◉

essential

Tyhjennä valinta

Does your paper address subitem 6a-ii?

Copy and paste relevant sections from manuscript text

"Parents randomized to the IG received instructions for downloading the free BuddyCare application (Android or iOS) and its activation code 3–4 weeks before their child's surgery. Alternatively, the parents could access a web-based application portal via a web browser. The application reminded users about important instructions to be followed two weeks before and one week after the surgery. Hospital staff assigned a start date, time limit, and expiration date for various tasks and instructions, based on their assessment of when particular information was relevant to the child and their family. The timeline employed color-coding and spacers to make it easy for both children and family members to comprehend. The family was also able to access information in different formats based on their preferences. All the children and parents undergoing the same surgery received the same information at the same time. Forms filled out through the application went directly to hospital staff. Hospital staff could monitor the utilization rate of the application throughout, but its contents were not modified during the study. No problems with the application were reported during the study period. Families who did not use the application were contacted directly. In this study, we utilized the TIDieR (the Template for Intervention Description and Replication) checklist and guide to ensure a clear description of the intervention implemented. Check tidierguide.org [47]."

6a-iii) Describe whether, how, and when qualitative feedback from participants was obtained

Describe whether, how, and when qualitative feedback from participants was obtained (e.g., through emails, feedback forms, interviews, focus groups).

subitem not at all important

1 ◯

2 ◯

3 ◯

4 ◯

5 ◉

essential

Tyhjennä valinta

Does your paper address subitem 6a-iii?

Copy and paste relevant sections from manuscript text

I am working on a new publication that showcases various user experiences.

6b) Any changes to trial outcomes after the trial commenced, with reasons

Does your paper address CONSORT subitem 6b? *

Copy and paste relevant sections from the manuscript (include quotes in quotation marks "like this" to indicate direct quotes from your manuscript), or elaborate on this item by providing additional information not in the ms, or briefly explain why the item is not applicable/relevant for your study

No changes to trial outcomes after the trial commenced

7a) How sample size was determined

NPT: When applicable, details of whether and how the clustering by care provides or centers was addressed

7a-i) Describe whether and how expected attrition was taken into account when calculating the sample size

Describe whether and how expected attrition was taken into account when calculating the sample size.

subitem not at all important

1 ○

2 ○

3 ○

4 ○

5 ◉

essential

Tyhjennä valinta

Does your paper address subitem 7a-i?

Copy and paste relevant sections from manuscript title (include quotes in quotation marks "like this" to indicate direct quotes from your manuscript), or elaborate on this item by providing additional information not in the ms, or briefly explain why the item is not applicable/relevant for your study

"According to a study by Kain et al. (2009), 46% of all parents experience anxiety before surgery, as measured by the State-Trait Anxiety Inventory (STAI). To determine our sample size, we used the findings from Kain et al.'s study, which had an intervention group with a mean of 39.7 and a standard deviation of 11.5, and a control group with a mean of 48.6 and a standard deviation of 13.1. Our study focused on the primary outcome of parental anxiety, as measured by the STAI scale. An independent samples t-test with a two-sided alpha value of 0.05 and 80% power estimated that 50 participants would be required for the study, with 25 participants in each group (intervention and control). We adjusted the sample size to account for a potential 30% drop-out rate. As a result, the final sample consisted of 71 participants, with 36 in the intervention group and 35 in the control group. A total of 71 parents were recruited and randomized [45]. "

7b) When applicable, explanation of any interim analyses and stopping guidelines

Does your paper address CONSORT subitem 7b? *

Copy and paste relevant sections from the manuscript (include quotes in quotation marks "like this" to indicate direct quotes from your manuscript), or elaborate on this item by providing additional information not in the ms, or briefly explain why the item is not applicable/relevant for your study

Oma vastauksesi

8a) Method used to generate the random allocation sequence

NPT: When applicable, how care providers were allocated to each trial group

Does your paper address CONSORT subitem 8a? *

Copy and paste relevant sections from the manuscript (include quotes in quotation marks "like this" to indicate direct quotes from your manuscript), or elaborate on this item by providing additional information not in the ms, or briefly explain why the item is not applicable/relevant for your study

"The eligible participants were stratified into five strata according to the age of the child undergoing surgery (2, 3, 4, 5, and 6 years), and then randomized into each group at a 1:1 ratio. We used stratified simple randomization to keep the groups as similar as possible [46]. The researcher prepared two envelopes in advance, with one for 2-year-olds and the other for children up to the age of six. A total of 10 notes were placed into each age group envelope, five of which were allocated into the intervention group (IG) and the remaining five allocated into the control group (CG). Following ethical guidelines, after a telephone conversation with each set of parents, the researcher took one note from the envelope corresponding to the child's age group to determine whether the family was allocated to the IG or CG. Both researcher and participants were unaware of which group they were allocated to prior to the study. A flow chart of the study is presented in Figure 1."

8b) Type of randomisation; details of any restriction (such as blocking and block size)

Does your paper address CONSORT subitem 8b? *

Copy and paste relevant sections from the manuscript (include quotes in quotation marks "like this" to indicate direct quotes from your manuscript), or elaborate on this item by providing additional information not in the ms, or briefly explain why the item is not applicable/relevant for your study

"The eligible participants were stratified into five strata according to the age of the child undergoing surgery (2, 3, 4, 5, and 6 years), and then randomized into each group at a 1:1 ratio. We used stratified simple randomization to keep the groups as similar as possible [46]. The researcher prepared two envelopes in advance, with one for 2-year-olds and the other for children up to the age of six. A total of 10 notes were placed into each age group envelope, five of which were allocated into the intervention group (IG) and the remaining five allocated into the control group (CG). Following ethical guidelines, after a telephone conversation with each set of parents, the researcher took one note from the envelope corresponding to the child's age group to determine whether the family was allocated to the IG or CG. Both researcher and participants were unaware of which group they were allocated to prior to the study. A flow chart of the study is presented in Figure 1."

9) Mechanism used to implement the random allocation sequence (such as sequentially numbered containers), describing any steps taken to conceal the sequence until interventions were assigned

Does your paper address CONSORT subitem 9? *

Copy and paste relevant sections from the manuscript (include quotes in quotation marks "like this" to indicate direct quotes from your manuscript), or elaborate on this item by providing additional information not in the ms, or briefly explain why the item is not applicable/relevant for your study

"The eligible participants were stratified into five strata according to the age of the child undergoing surgery (2, 3, 4, 5, and 6 years), and then randomized into each group at a 1:1 ratio. We used stratified simple randomization to keep the groups as similar as possible [46]. The researcher prepared two envelopes in advance, with one for 2-year-olds and the other for children up to the age of six. A total of 10 notes were placed into each age group envelope, five of which were allocated into the intervention group (IG) and the remaining five allocated into the control group (CG). Following ethical guidelines, after a telephone conversation with each set of parents, the researcher took one note from the envelope corresponding to the child's age group to determine whether the family was allocated to the IG or CG. Both researcher and participants were unaware of which group they were allocated to prior to the study. A flow chart of the study is presented in Figure 1."

10) Who generated the random allocation sequence, who enrolled participants, and who assigned participants to interventions

Does your paper address CONSORT subitem 10? *

Copy and paste relevant sections from the manuscript (include quotes in quotation marks "like this" to indicate direct quotes from your manuscript), or elaborate on this item by providing additional information not in the ms, or briefly explain why the item is not applicable/relevant for your study

"The eligible participants were stratified into five strata according to the age of the child undergoing surgery (2, 3, 4, 5, and 6 years), and then randomized into each group at a 1:1 ratio. We used stratified simple randomization to keep the groups as similar as possible [46]. The researcher prepared two envelopes in advance, with one for 2-year-olds and the other for children up to the age of six. A total of 10 notes were placed into each age group envelope, five of which were allocated into the intervention group (IG) and the remaining five allocated into the control group (CG). Following ethical guidelines, after a telephone conversation with each set of parents, the researcher took one note from the envelope corresponding to the child's age group to determine whether the family was allocated to the IG or CG. Both researcher and participants were unaware of which group they were allocated to prior to the study. A flow chart of the study is presented in Figure 1."

11a) If done, who was blinded after assignment to interventions (for example, participants, care providers, those assessing outcomes) and how

NPT: Whether or not administering co-interventions were blinded to group assignment

11a-i) Specify who was blinded, and who wasn't

Specify who was blinded, and who wasn't. Usually, in web-based trials it is not possible to blind the participants [1, 3] (this should be clearly acknowledged), but it may be possible to blind outcome assessors, those doing data analysis or those administering co-interventions (if any).

subitem not at all important

1 ○

2 ○

3 ○

4 ○

5 ◉

essential

Tyhjennä valinta

Does your paper address subitem 11a-i? *

Copy and paste relevant sections from the manuscript (include quotes in quotation marks "like this" to indicate direct quotes from your manuscript), or elaborate on this item by providing additional information not in the ms, or briefly explain why the item is not applicable/relevant for your study

"The study design also made blinding difficult. It was impossible to blind the nurses because they needed to know which parents were in the CG so that they could call them the day before the surgery."

11a-ii) Discuss e.g., whether participants knew which intervention was the "intervention of interest" and which one was the "comparator"

Informed consent procedures (4a-ii) can create biases and certain expectations - discuss e.g., whether participants knew which intervention was the "intervention of interest" and which one was the "comparator".

subitem not at all important

1 ○

2 ○

3 ○

4 ○

5 ◉

essential

Tyhjennä valinta

Does your paper address subitem 11a-ii?

Copy and paste relevant sections from the manuscript (include quotes in quotation marks "like this" to indicate direct quotes from your manuscript), or elaborate on this item by providing additional information not in the ms, or briefly explain why the item is not applicable/relevant for your study

"When a surgery was confirmed, hospital staff added the child, surgery type, and schedule into the application. The application then automatically selected the material suitable to that child and family. Hospital staff could monitor the family's use of the application and receive and accept completed forms through the application. The application also reminded the family about how to tell the child about the surgery and offered other preparation instructions essential to the operation. The mobile application contained all the necessary information about the child's surgery and made it possible to complete pre-information forms. It enabled parents to access information at convenient times whether they were at home, at work, travelling or during their free time. Parents also had the option of getting in touch with the hospital if problems arose with the application.

Parents randomized to the IG received instructions for downloading the free BuddyCare application (Android or iOS) and its activation code 3–4 weeks before their child's surgery. "

11b) If relevant, description of the similarity of interventions
(this item is usually not relevant for ehealth trials as it refers to similarity of a placebo or sham intervention to a active medication/intervention)

Does your paper address CONSORT subitem 11b? *

Copy and paste relevant sections from the manuscript (include quotes in quotation marks "like this" to indicate direct quotes from your manuscript), or elaborate on this item by providing additional information not in the ms, or briefly explain why the item is not applicable/relevant for your study

"The CG received routine preparation while the IG was prepared using a mobile [illegible]

12a) Statistical methods used to compare groups for primary and secondary outcomes
NPT: When applicable, details of whether and how the clustering by care providers or centers was addressed

Does your paper address CONSORT subitem 12a? *

Copy and paste relevant sections from the manuscript (include quotes in quotation marks "like this" to indicate direct quotes from your manuscript), or elaborate on this item by providing additional information not in the ms, or briefly explain why the item is not applicable/relevant for your study

"Descriptive statistics (frequencies with percentages, medians with interquartile range (IQR) and means with standard deviation (SD)) were used to express the parents' characteristics and study variables. The analyses were performed using IBM SPSS statistical software for Windows (version 28; SPSS Inc., Chicago, IL). "

12a-i) Imputation techniques to deal with attrition / missing values

Imputation techniques to deal with attrition / missing values: Not all participants will use the intervention/comparator as intended and attrition is typically high in ehealth trials. Specify how participants who did not use the application or dropped out from the trial were treated in the statistical analysis (a complete case analysis is strongly discouraged, and simple imputation techniques such as LOCF may also be problematic [4]).

subitem not at all important

1 ○

2 ○

3 ○

4 ○

5 ◉

essential

Tyhjennä valinta

Does your paper address subitem 12a-i? *

Copy and paste relevant sections from the manuscript (include quotes in quotation marks "like this" to indicate direct quotes from your manuscript), or elaborate on this item by providing additional information not in the ms, or briefly explain why the item is not applicable/relevant for your study

"The resulting missing values could not be substituted with mean values due to the small size of the sample."

12b) Methods for additional analyses, such as subgroup analyses and adjusted analyses

Does your paper address CONSORT subitem 12b? *

Copy and paste relevant sections from the manuscript (include quotes in quotation marks "like this" to indicate direct quotes from your manuscript), or elaborate on this item by providing additional information not in the ms, or briefly explain why the item is not applicable/relevant for your study

"Descriptive statistics (frequencies with percentages, medians with interquartile range (IQR) and means with standard deviation (SD)) were used to express the parents' characteristics and study variables. The distributions of the measured variables were verified. The anxiety levels of the parents were found to have a normal distribution, and parametric tests were used to examine the results. However, the stress levels of the parents, as well as the pain and fear levels of the children, had abnormal distributions. As a result, non-parametric tests were utilized to analyze those variables. Intention-to-treat analysis was performed, which meant that all parents with children participating in the study were analyzed in the groups to which they were initially randomized. Parents' stress was re-categorized into three categories: no stress (VRSS = 0), mild stress (1) and moderate to intense stress (2–5). Child's pain, assessed by the child, was re-categorized into three categories: no pain (WBS = 0), moderate pain (2–4), and severe pain (6–10). The significance of between-group differences in the variables measured was assessed using the t-test (S-Anxiety sum score), Chi-square test (categorized study variables), or Mann-Whitney U-test (child's pain, assessed by parents and nurse (VAS) and child's fear (FAS)). Changes between each assessment were analyzed separately for IG and CG using paired samples t-test (S-Anxiety sum score), sign-test (categorized study variables) or Wilcoxon signed-rank test (child's pain, assessed by parents and child's fear) with Benjamini-Hochberg correction with false positive rate of 0.05 for multiple comparisons. In the analysis of change, data from those who replied for all assessments were used (T1, T2, T3 and T4). The threshold for statistical significance was set at p-value≤0.05. The analyses were performed using IBM SPSS statistical software for Windows (version 28; SPSS Inc., Chicago, IL). "

X26) REB/IRB Approval and Ethical Considerations [recommended as subheading under "Methods"] (not a CONSORT item)

X26-i) Comment on ethics committee approval

subitem not at all important

1 ○

2 ○

3 ○

4 ○

5 ◉

essential

Tyhjennä valinta

Does your paper address subitem X26-i?

Copy and paste relevant sections from the manuscript (include quotes in quotation marks "like this" to indicate direct quotes from your manuscript), or elaborate on this item by providing additional information not in the ms, or briefly explain why the item is not applicable/relevant for your study

This study received ethical approval from the Northern Ostrobothnia Regional Ethics Committee Board (EETTMK:53/2017) in June 2017.

x26-ii) Outline informed consent procedures

Outline informed consent procedures e.g., if consent was obtained offline or online (how? Checkbox, etc.?), and what information was provided (see 4a-ii). See [6] for some items to be included in informed consent documents.

subitem not at all important

1 ○

2 ○

3 ○

4 ○

5 ◉

essential

Tyhjennä valinta

Does your paper address subitem X26-ii?

Copy and paste relevant sections from the manuscript (include quotes in quotation marks "like this" to indicate direct quotes from your manuscript), or elaborate on this item by providing additional information not in the ms, or briefly explain why the item is not applicable/relevant for your study

The parents were informed about the study both verbally and in writing.

X26-iii) Safety and security procedures

Safety and security procedures, incl. privacy considerations, and any steps taken to reduce the likelihood or detection of harm (e.g., education and training, availability of a hotline)

subitem not at all important

1 ○

2 ○

3 ○

4 ○

5 ◉

essential

Tyhjennä valinta

Does your paper address subitem X26-iii?

Copy and paste relevant sections from the manuscript (include quotes in quotation marks "like this" to indicate direct quotes from your manuscript), or elaborate on this item by providing additional information not in the ms, or briefly explain why the item is not applicable/relevant for your study

"Ethical considerations were respected at all stages of the study, including the voluntary participation of family members, the right to information about the research before and throughout data collection, the right to ask questions, the right to be treated with respect and honesty, the right to suspend research, and the right to ask questions. Subjects' privacy and data protection were ensured throughout the data collection process [60]."

RESULTS

13a) For each group, the numbers of participants who were randomly assigned, received intended treatment, and were analysed for the primary outcome

NPT: The number of care providers or centers performing the intervention in each group and the number of patients treated by each care provider in each center

Does your paper address CONSORT subitem 13a? *

Copy and paste relevant sections from the manuscript (include quotes in quotation marks "like this" to indicate direct quotes from your manuscript), or elaborate on this item by providing additional information not in the ms, or briefly explain why the item is not applicable/relevant for your study

"A total of 71 children and their parents were recruited for the study. The IG comprised 36 participants (loss rate 22.2 %) and the CG 34 (loss rate 23.5 %). A total of 54 responses from participants in the IG (n = 28) and CG (n = 26) were analyzed (participation rate 76.1 %) (Figure 1)."

13b) For each group, losses and exclusions after randomisation, together with reasons

Does your paper address CONSORT subitem 13b? (NOTE: Preferably, this is shown in a CONSORT flow diagram) *

Copy and paste relevant sections from the manuscript (include quotes in quotation marks "like this" to indicate direct quotes from your manuscript), or elaborate on this item by providing additional information not in the ms, or briefly explain why the item is not applicable/relevant for your study

"The reporting of the study results conforms with the CONSORT Statement [59]"

13b-i) Attrition diagram

Strongly recommended: An attrition diagram (e.g., proportion of participants still logging in or using the intervention/comparator in each group plotted over time, similar to a survival curve) or other figures or tables demonstrating usage/dose/engagement.

subitem not at all important

1 ○

2 ○

3 ○

4 ○

5 ◉

essential

Tyhjennä valinta

Does your paper address subitem 13b-i?

Copy and paste relevant sections from the manuscript or cite the figure number if applicable (include quotes in quotation marks "like this" to indicate direct quotes from your manuscript), or elaborate on this item by providing additional information not in the ms, or briefly explain why the item is not applicable/relevant for your study

"The reporting of the study results conforms with the CONSORT Statement [59]"

14a) Dates defining the periods of recruitment and follow-up

Does your paper address CONSORT subitem 14a? *

Copy and paste relevant sections from the manuscript (include quotes in quotation marks "like this" to indicate direct quotes from your manuscript), or elaborate on this item by providing additional information not in the ms, or briefly explain why the item is not applicable/relevant for your study

"The reporting of the study results conforms with the CONSORT Statement [59]"

14a-i) Indicate if critical “secular events” fell into the study period

Indicate if critical “secular events” fell into the study period, e.g., significant changes in Internet resources available or “changes in computer hardware or Internet delivery resources”

subitem not at all important

1 ○

2 ○

3 ○

4 ○

5 ◉

essential

Tyhjennä valinta

Does your paper address subitem 14a-i?

Copy and paste relevant sections from the manuscript (include quotes in quotation marks "like this" to indicate direct quotes from your manuscript), or elaborate on this item by providing additional information not in the ms, or briefly explain why the item is not applicable/relevant for your study

The event in question did not occur.

14b) Why the trial ended or was stopped (early)

Does your paper address CONSORT subitem 14b? *

Copy and paste relevant sections from the manuscript (include quotes in quotation marks "like this" to indicate direct quotes from your manuscript), or elaborate on this item by providing additional information not in the ms, or briefly explain why the item is not applicable/relevant for your study

The event in question did not occur.

15) A table showing baseline demographic and clinical characteristics for each group

NPT: When applicable, a description of care providers (case volume, qualification, expertise, etc.) and centers (volume) in each group

Does your paper address CONSORT subitem 15? *

Copy and paste relevant sections from the manuscript (include quotes in quotation marks "like this" to indicate direct quotes from your manuscript), or elaborate on this item by providing additional information not in the ms, or briefly explain why the item is not applicable/relevant for your study

"The reporting of the study results conforms with the CONSORT Statement [59]"

15-i) Report demographics associated with digital divide issues

In ehealth trials it is particularly important to report demographics associated with digital divide issues, such as age, education, gender, social-economic status, computer/Internet/ehealth literacy of the participants, if known.

subitem not at all important

1 ○

2 ○

3 ○

4 ○

5 ◉

essential

Tyhjennä valinta

Does your paper address subitem 15-i? *

Copy and paste relevant sections from the manuscript (include quotes in quotation marks "like this" to indicate direct quotes from your manuscript), or elaborate on this item by providing additional information not in the ms, or briefly explain why the item is not applicable/relevant for your study

"Table 2. Comparison of baseline demographic information between the intervention (n=28) and control (n=26) groups."

16) For each group, number of participants (denominator) included in each analysis and whether the analysis was by original assigned groups

16-i) Report multiple “denominators” and provide definitions

Report multiple “denominators” and provide definitions: Report N’s (and effect sizes) “across a range of study participation [and use] thresholds” [1], e.g., N exposed, N consented, N used more than x times, N used more than y weeks, N participants “used” the intervention/comparator at specific pre-defined time points of interest (in absolute and relative numbers per group). Always clearly define “use” of the intervention.

subitem not at all important

1 ○

2 ○

3 ○

4 ○

5 ◉

essential

Tyhjennä valinta

Does your paper address subitem 16-i? *

Copy and paste relevant sections from the manuscript (include quotes in quotation marks "like this" to indicate direct quotes from your manuscript), or elaborate on this item by providing additional information not in the ms, or briefly explain why the item is not applicable/relevant for your study

"Descriptive statistics (frequencies with percentages, medians with interquartile range (IQR) and means with standard deviation (SD)) were used to express the parents' characteristics and study variables. The distributions of the measured variables were verified. The anxiety levels of the parents were found to have a normal distribution, and parametric tests were used to examine the results. However, the stress levels of the parents, as well as the pain and fear levels of the children, had abnormal distributions. As a result, non-parametric tests were utilized to analyze those variables. Intention-to-treat analysis was performed, which meant that all parents with children participating in the study were analyzed in the groups to which they were initially randomized.

The significance of between-group differences in the variables measured was assessed using the t-test (S-Anxiety sum score), Chi-square test (categorized study variables), or Mann-Whitney U-test (child's pain, assessed by parents and nurse (VAS) and child's fear (FAS)). Changes between each assessment were analyzed separately for IG and CG using paired samples t-test (S-Anxiety sum score), sign-test (categorized study variables) or Wilcoxon signed-rank test (child's pain, assessed by parents and child's fear) with Benjamini-Hochberg correction with false positive rate of 0.05 for multiple comparisons. In the analysis of change, data from those who replied for all assessments were used (T1, T2, T3 and T4). The threshold for statistical significance was set at p-value≤0.05. The analyses were performed using IBM SPSS statistical software for Windows (version 28; SPSS Inc., Chicago, IL). Effect sizes were calculated. Appendix 2.

16-ii) Primary analysis should be intent-to-treat

Primary analysis should be intent-to-treat, secondary analyses could include comparing only “users”, with the appropriate caveats that this is no longer a randomized sample (see 18-i).

subitem not at all important

1 ○

2 ○

3 ○

4 ○

5 ◉

essential

Tyhjennä valinta

Does your paper address subitem 16-ii?

Copy and paste relevant sections from the manuscript (include quotes in quotation marks "like this" to indicate direct quotes from your manuscript), or elaborate on this item by providing additional information not in the ms, or briefly explain why the item is not applicable/relevant for your study

"Intention-to-treat analysis was performed, which meant that all parents with children participating in the study were analyzed in the groups to which they were initially randomized."

17a) For each primary and secondary outcome, results for each group, and the estimated effect size and its precision (such as 95% confidence interval)

Does your paper address CONSORT subitem 17a? *

Copy and paste relevant sections from the manuscript (include quotes in quotation marks "like this" to indicate direct quotes from your manuscript), or elaborate on this item by providing additional information not in the ms, or briefly explain why the item is not applicable/relevant for your study

"Overall, parental anxiety decreased from the preoperative assessment (mean 36.3; SD 10.3) to the postoperative assessment (mean 29.4; SD 6.9) the average change being -7.0 (95% CI -9.8 to -4.1; P < 0.001). Separately, within group changes were -7.0 (95% CI -11.3: -2.6) in the IG and -7.0 (-11.0; -3.0) in the CG. (Figure 3)."

17a-i) Presentation of process outcomes such as metrics of use and intensity of use

In addition to primary/secondary (clinical) outcomes, the presentation of process outcomes such as metrics of use and intensity of use (dose, exposure) and their operational definitions is critical. This does not only refer to metrics of attrition (13-b) (often a binary variable), but also to more continuous exposure metrics such as "average session length". These must be accompanied by a technical description how a metric like a "session" is defined (e.g., timeout after idle time) [1] (report under item 6a).

subitem not at all important

1 ○

2 ○

3 ○

4 ○

5 ◉

essential

Tyhjennä valinta

Does your paper address subitem 17a-i?

Copy and paste relevant sections from the manuscript (include quotes in quotation marks "like this" to indicate direct quotes from your manuscript), or elaborate on this item by providing additional information not in the ms, or briefly explain why the item is not applicable/relevant for your study

The results of the process, such as usage metrics and usage intensity, were described with different parameters.

17b) For binary outcomes, presentation of both absolute and relative effect sizes is recommended

Does your paper address CONSORT subitem 17b? *

Copy and paste relevant sections from the manuscript (include quotes in quotation marks "like this" to indicate direct quotes from your manuscript), or elaborate on this item by providing additional information not in the ms, or briefly explain why the item is not applicable/relevant for your study

Oma vastauksesi

18) Results of any other analyses performed, including subgroup analyses and adjusted analyses, distinguishing pre-specified from exploratory

Does your paper address CONSORT subitem 18? *

Copy and paste relevant sections from the manuscript (include quotes in quotation marks "like this" to indicate direct quotes from your manuscript), or elaborate on this item by providing additional information not in the ms, or briefly explain why the item is not applicable/relevant for your study

The study presented additional analyses, including subgroup and adjusted analyses, categorized as pre-specified or exploratory.

18-i) Subgroup analysis of comparing only users

A subgroup analysis of comparing only users is not uncommon in ehealth trials, but if done, it must be stressed that this is a self-selected sample and no longer an unbiased sample from a randomized trial (see 16-iii).

subitem not at all important

1 ◯

2 ◯

3 ◯

4 ◯

5 ◉

essential

Tyhjennä valinta

Does your paper address subitem 18-i?

Copy and paste relevant sections from the manuscript (include quotes in quotation marks "like this" to indicate direct quotes from your manuscript), or elaborate on this item by providing additional information not in the ms, or briefly explain why the item is not applicable/relevant for your study

The presented findings are based on a randomized trial and are clearly displayed.

19) All important harms or unintended effects in each group

(for specific guidance see CONSORT for harms)

Does your paper address CONSORT subitem 19? *

Copy and paste relevant sections from the manuscript (include quotes in quotation marks "like this" to indicate direct quotes from your manuscript), or elaborate on this item by providing additional information not in the ms, or briefly explain why the item is not applicable/relevant for your study

The limitations of the study were presented honestly.

19-i) Include privacy breaches, technical problems

Include privacy breaches, technical problems. This does not only include physical “harm” to participants, but also incidents such as perceived or real privacy breaches [1], technical problems, and other unexpected/unintended incidents. “Unintended effects” also includes unintended positive effects [2].

subitem not at all important

1 ○

2 ○

3 ○

4 ○

5 ◉

essential

Tyhjennä valinta

Does your paper address subitem 19-i?

Copy and paste relevant sections from the manuscript (include quotes in quotation marks "like this" to indicate direct quotes from your manuscript), or elaborate on this item by providing additional information not in the ms, or briefly explain why the item is not applicable/relevant for your study

"Parents also had the option of getting in touch with the hospital if problems arose with the application."

19-ii) Include qualitative feedback from participants or observations from staff/researchers

Include qualitative feedback from participants or observations from staff/researchers, if available, on strengths and shortcomings of the application, especially if they point to unintended/unexpected effects or uses. This includes (if available) reasons for why people did or did not use the application as intended by the developers.

subitem not at all important

1 ○

2 ○

3 ○

4 ○

5 ◉

essential

Tyhjennä valinta

Does your paper address subitem 19-ii?

Copy and paste relevant sections from the manuscript (include quotes in quotation marks "like this" to indicate direct quotes from your manuscript), or elaborate on this item by providing additional information not in the ms, or briefly explain why the item is not applicable/relevant for your study

Another publication will be released in relation to this matter.

DISCUSSION

22) Interpretation consistent with results, balancing benefits and harms, and considering other relevant evidence

NPT: In addition, take into account the choice of the comparator, lack of or partial blinding, and unequal expertise of care providers or centers in each group

22-i) Restate study questions and summarize the answers suggested by the data, starting with primary outcomes and process outcomes (use)

Restate study questions and summarize the answers suggested by the data, starting with primary outcomes and process outcomes (use).

subitem not at all important

1 ○

2 ○

3 ○

4 ○

5 ◉

essential

Tyhjennä valinta

Does your paper address subitem 22-i? *

Copy and paste relevant sections from the manuscript (include quotes in quotation marks "like this" to indicate direct quotes from your manuscript), or elaborate on this item by providing additional information not in the ms, or briefly explain why the item is not applicable/relevant for your study

The analysis is coherent with the findings, taking into account both advantages and disadvantages.

22-ii) Highlight unanswered new questions, suggest future research

Highlight unanswered new questions, suggest future research.

subitem not at all important

1 ○

2 ○

3 ○

4 ○

5 ◉

essential

Tyhjennä valinta

Does your paper address subitem 22-ii?

Copy and paste relevant sections from the manuscript (include quotes in quotation marks "like this" to indicate direct quotes from your manuscript), or elaborate on this item by providing additional information not in the ms, or briefly explain why the item is not applicable/relevant for your study

The importance of researching the topic is rightly highlighted

20) Trial limitations, addressing sources of potential bias, imprecision, and, if relevant, multiplicity of analyses

20-i) Typical limitations in ehealth trials

Typical limitations in ehealth trials: Participants in ehealth trials are rarely blinded. Ehealth trials often look at a multiplicity of outcomes, increasing risk for a Type I error. Discuss biases due to non-use of the intervention/usability issues, biases through informed consent procedures, unexpected events.

subitem not at all important

1 ○

2 ○

3 ○

4 ○

5 ◉

essential

Tyhjennä valinta

Does your paper address subitem 20-i? *

Copy and paste relevant sections from the manuscript (include quotes in quotation marks "like this" to indicate direct quotes from your manuscript), or elaborate on this item by providing additional information not in the ms, or briefly explain why the item is not applicable/relevant for your study

It is of utmost importance to tackle any potential biases that could arise due to the trial's limitations.This is also how we do it.

21) Generalisability (external validity, applicability) of the trial findings

NPT: External validity of the trial findings according to the intervention, comparators, patients, and care providers or centers involved in the trial

21-i) Generalizability to other populations

Generalizability to other populations: In particular, discuss generalizability to a general Internet population, outside of a RCT setting, and general patient population, including applicability of the study results for other organizations

subitem not at all important

1 ○

2 ○

3 ○

4 ○

5 ◉

essential

Tyhjennä valinta

Does your paper address subitem 21-i?

Copy and paste relevant sections from the manuscript (include quotes in quotation marks "like this" to indicate direct quotes from your manuscript), or elaborate on this item by providing additional information not in the ms, or briefly explain why the item is not applicable/relevant for your study

Although the sample size is small, the results can serve as a starting point for a follow-up study. However, the generalizability of these findings may be uncertain.

21-ii) Discuss if there were elements in the RCT that would be different in a routine application setting

Discuss if there were elements in the RCT that would be different in a routine application setting (e.g., prompts/reminders, more human involvement, training sessions or other co-interventions) and what impact the omission of these elements could have on use, adoption, or outcomes if the intervention is applied outside of a RCT setting.

subitem not at all important

1 ○

2 ○

3 ○

4 ○

5 ◉

essential

Tyhjennä valinta

Does your paper address subitem 21-ii?

Copy and paste relevant sections from the manuscript (include quotes in quotation marks "like this" to indicate direct quotes from your manuscript), or elaborate on this item by providing additional information not in the ms, or briefly explain why the item is not applicable/relevant for your study

These are taken into account.

OTHER INFORMATION

23) Registration number and name of trial registry

Does your paper address CONSORT subitem 23? *

Copy and paste relevant sections from the manuscript (include quotes in quotation marks "like this" to indicate direct quotes from your manuscript), or elaborate on this item by providing additional information not in the ms, or briefly explain why the item is not applicable/relevant for your study

"This study was registered at ClinicalTrials.gov (NCT03774303)."

24) Where the full trial protocol can be accessed, if available

Does your paper address CONSORT subitem 24? *

Cite a Multimedia Appendix, other reference, or copy and paste relevant sections from the manuscript (include quotes in quotation marks "like this" to indicate direct quotes from your manuscript), or elaborate on this item by providing additional information not in the ms, or briefly explain why the item is not applicable/relevant for your study

"This study was registered at ClinicalTrials.gov (NCT03774303)."

25) Sources of funding and other support (such as supply of drugs), role of funders

Does your paper address CONSORT subitem 25? *

Copy and paste relevant sections from the manuscript (include quotes in quotation marks "like this" to indicate direct quotes from your manuscript), or elaborate on this item by providing additional information not in the ms, or briefly explain why the item is not applicable/relevant for your study

"This research has received grants from the Research Foundation of the Mannerheim Child Protection Union, the Pediatric Research Foundation and the Society for Research in Nursing Sciences (HTTS)."

X27) Conflicts of Interest (not a CONSORT item)

X27-i) State the relation of the study team towards the system being evaluated

In addition to the usual declaration of interests (financial or otherwise), also state the relation of the study team towards the system being evaluated, i.e., state if the authors/evaluators are distinct from or identical with the developers/sponsors of the intervention.

subitem not at all important

1 ○

2 ○

3 ○

4 ○

5 ◉

essential

Tyhjennä valinta

Does your paper address subitem X27-i?

Copy and paste relevant sections from the manuscript (include quotes in quotation marks "like this" to indicate direct quotes from your manuscript), or elaborate on this item by providing additional information not in the ms, or briefly explain why the item is not applicable/relevant for your study

CRediT authorship contribution statement

About the CONSORT EHEALTH checklist

As a result of using this checklist, did you make changes in your manuscript? *

○ yes, major changes

○ yes, minor changes

◉ no

What were the most important changes you made as a result of using this checklist?

Oma vastauksesi

How much time did you spend on going through the checklist INCLUDING making changes in your manuscript *

I spent about one hour going through the checklist.

As a result of using this checklist, do you think your manuscript has improved? *

- ○ yes
- ◉ no
- ○ Muu:

Would you like to become involved in the CONSORT EHEALTH group?

This would involve for example becoming involved in participating in a workshop and writing an "Explanation and Elaboration" document

- ○ yes
- ◉ no
- ○ Muu:

Tyhjennä valinta

Any other comments or questions on CONSORT EHEALTH

Oma vastauksesi

STOP - Save this form as PDF before you click submit

To generate a record that you filled in this form, we recommend to generate a PDF of this page (on a Mac, simply select "print" and then select "print as PDF") before you submit it.

When you submit your (revised) paper to JMIR, please upload the PDF as supplementary file.

Don't worry if some text in the textboxes is cut off, as we still have the complete information in our database. Thank you!

Final step: Click submit !

Click submit so we have your answers in our database!

Lähetä

Tyhjennä lomake

Älä koskaan lähetä salasanaa Google Formsin kautta.

Google ei ole luonut tai hyväksynyt tätä sisältöä. Ilmoita väärinkäytöstä - Palveluehdot - Tietosuojakäytäntö

Google Forms
